# Acid sphingomyelinase regulates the localization and trafficking of palmitoylated proteins

**DOI:** 10.1242/bio.040311

**Published:** 2019-05-29

**Authors:** Xiahui Xiong, Chia-Fang Lee, Wenjing Li, Jiekai Yu, Linyu Zhu, Yongsoon Kim, Hui Zhang, Hong Sun

**Affiliations:** 1 Department of Chemistry and Biochemistry, University of Nevada, Las Vegas, Las Vegas, NV 89154-4003, USA; 2 Protea Biosciences, 1311 Pineview drive, Morgantown, West Virginia, USA

**Keywords:** Acid sphingomyelinase, Ceramide, Lipid raft, Proteomics, Protein palmitoylation, Protein trafficking, Plasma membrane, Golgi

## Abstract

In human, loss of acid sphingomyelinase (ASM/SMPD1) causes Niemann–Pick disease, type A. ASM hydrolyzes sphingomyelins to produce ceramides but protein targets of ASM remain largely unclear. Our mass spectrometry-based proteomic analyses have identified >100 proteins associated with the ASM-dependent, detergent-resistant membrane microdomains (lipid rafts), with >60% of these proteins being palmitoylated, including SNAP23, Src-family kinases Yes and Lyn, and Ras and Rab family small GTPases. Inactivation of ASM abolished the presence of these proteins in the plasma membrane, with many of them trapped in the Golgi. While palmitoylation inhibitors and palmitoylation mutants phenocopied the effects of ASM inactivation, we demonstrated that ASM is required for the transport of palmitoylated proteins, such as SNAP23 and Lyn, from the Golgi to the plasma membrane without affecting palmitoylation directly. Importantly, ASM delivered extracellularly can regulate the trafficking of SNAP23 from the Golgi to the plasma membrane. Our studies suggest that ASM, acting at the plasma membrane to produce ceramides, regulates the localization and trafficking of the palmitoylated proteins.

## Abstract (precis)

**Summary:** Acid sphingomyelinase (ASM) regulates palmitoylated protein trafficking and localization.

## INTRODUCTION

The plasma membrane is a lipid bilayer composed primarily of phospholipids, as well as sphingomyelins, cholesterol, glycol-sphingolipids and other less abundant lipid molecules such as ceramides (Holthuis and Menon, 2014). Sphingomyelins comprise about 10–20% total lipids and are asymmetrically localized in the outer leaflet of the lipid bilayer of the plasma membrane (Holthuis and Menon, 2014). Sphingomyelins can also interact with cholesterol through their acyl tails. The tightly packing of sphingomyelins and cholesterol can form an ordered lipid microdomain, the lipid raft (Simons and Ikonen, 1997). Increasing evidence suggests that lipid rafts can serve as signaling platforms to facilitate protein–protein interactions, as demonstrated for the activation of T cell receptor (Lingwood and Simons, 2010; Rajendran and Simons, 2005). Sphingomyelins can be converted to ceramides through the action of sphingomyelinases. Ceramides, composed of a sphingosine and a fatty acid, are much more hydrophobic than sphingomyelin. Ceramides can self-associate to form unique lipid microdomains in artificial membrane or in the plasma membrane of erythrocytes (Holopainen et al., 1998; Lopez-Montero et al., 2010; van Blitterswijk et al., 2003).

Acid sphingomyelinase (ASM) catalyzes the hydrolysis of sphingomyelins to produce ceramides and phosphocholine (Jenkins et al., 2009; Schuchman, 2007). In humans, loss-of-function mutations in the *ASM* gene (also called *SMPD1*) cause the familial Niemann–Pick disease, type A, with severe neurological deterioration and lysosomal accumulation of excessive sphingomyelins in brain, liver, spleen and lung cells, leading to the death of affected individuals at 1 or 2 years of age (Schuchman, 2007). Biochemically, ASM acts as a sphingomyelinase that catalyzes the hydrolysis of sphingomyelins to produce ceramides and phosphocholine. The precursors of sphingomyelin are first synthesized from ceramides in the endoplasmic reticulum (ER), transported to the Golgi apparatus and converted to sphingomyelins by sphingomyelin synthase 1 and 2 (SMS1 and 2) (Huitema et al., 2004). Sphingomyelin is then transported to the outer leaflet of the plasma lipid bilayer membrane. ASM, containing a saposin-like domain that is likely involved in binding to sphingomyelins (Jenkins et al., 2009), is also exported, likely from lysosomes, to the outer leaflet of plasma membrane to hydrolyze sphingomyelins into ceramides, which can form the ceramide-enriched lipid rafts in response to stress stimuli (Cremesti et al., 2001; Grassmé et al., 2003; Lopez-Montero et al., 2010; Tam et al., 2010; van Blitterswijk et al., 2003). Under normal conditions, the ratio of ceramides to sphingomyelins on the plasma membrane is usually low (1–5%) (Lopez-Montero et al., 2010; van Blitterswijk et al., 2003). However, in the ASM knockout mice, many pathological defects of Niemann–Pick Type A diseases were reproduced, including the extensive accumulation of sphingomyelins in liver, spleen, lung and brain cells (Horinouchi et al., 1995; Otterbach and Stoffel, 1995). These genetic studies demonstrate that ASM is a dynamic and critical regulator of sphingomyelin homeostasis in the plasma membrane. However, the physiological function of ASM and the critical protein targets regulated by ASM remain unclear.

Palmitoylation is a post-translational modification of proteins that involves the covalent attachment of saturated fatty acids, predominantly the C16:0 palmitate, to cysteine residues via a thioester linkage (Charollais and Van Der Goot, 2009; Resh, 2013; Salaun et al., 2010). Palmitoylation tethers the otherwise cytosolic proteins to the inner leaflets of the plasma membrane to facilitate the lateral diffusion of proteins in the plasma membrane and to promote protein–protein interaction, and is critical for signal transduction, synaptic function, membrane trafficking and vesicle fusion (Bijlmakers and Marsh, 2003; Smotrys and Linder, 2004). Many proteins, including the Src family tyrosine kinases such as Yes and Lyn, membrane trafficking proteins such as SNARE (soluble NSF attachment protein receptor) proteins, Ras family of small GTPases, receptors and channel proteins, are modified by palmitate to regulate their membrane-associated activities. Although palmitoylated proteins have been reported to be associated with lipid rafts in a manner that requires cholesterol participation (Chakrabandhu et al., 2007; Levental et al., 2010; Melkonian et al., 1999), the roles of ceramides in such association remain undetermined.

We have recently conducted a functional genome-wide screen in *Caenorhabditis* *elegans* and identified the worm homolog of ASM, *asm-3*, as a positive and novel regulator of the evolutionarily conserved IGF-1 receptor (IGF-1R)-like signaling pathway (Kim and Sun, 2007, 2012). Our recent studies in mammalian cells have shown that human ASM indeed functions to regulate the receptor tyrosine kinase signaling pathways such as the Met tyrosine kinase signaling (Zhu et al., 2016). In this report, we have used semi-quantitative proteomic approaches to identify proteins associated with lipid rafts that are regulated by ASM in human cells. Our studies have revealed novel function of ASM in regulation of intracellular protein localization and trafficking.

## RESULTS

### Using a biochemical procedure to fractionate ASM-regulated membrane-associated proteins

It has been shown that activation of CD95 death receptor in lymphoid cells is accompanied by the CD95 receptor localization into the ceramide-rich lipid rafts, and such localization is ASM-dependent, leading to CD95 receptor oligomerization, signaling and apoptosis (Cremesti et al., 2001; Lopez-Montero et al., 2010; van Blitterswijk et al., 2003). However, our recent genetic studies have shown that the *C. elegans* homolog of ASM is a positive regulator of the conserved IGF-1R-like signaling pathway *in vivo* (Kim and Sun, 2007, 2012). We wondered if ASM might regulate the localization of proteins in the ceramide-rich lipid rafts, and whether these proteins might be positively involved in receptor tyrosine kinase signaling under physiological conditions. To identify such proteins, we took a biochemical approach to isolate lipid rafts and analyze the associated proteins by mass-spectrometry. By comparing the lipid raft proteomes identified in cells with ASM or without ASM, we aim to identify the lipid raft-associated proteins that are regulated by ASM.

**Fig. 1.**
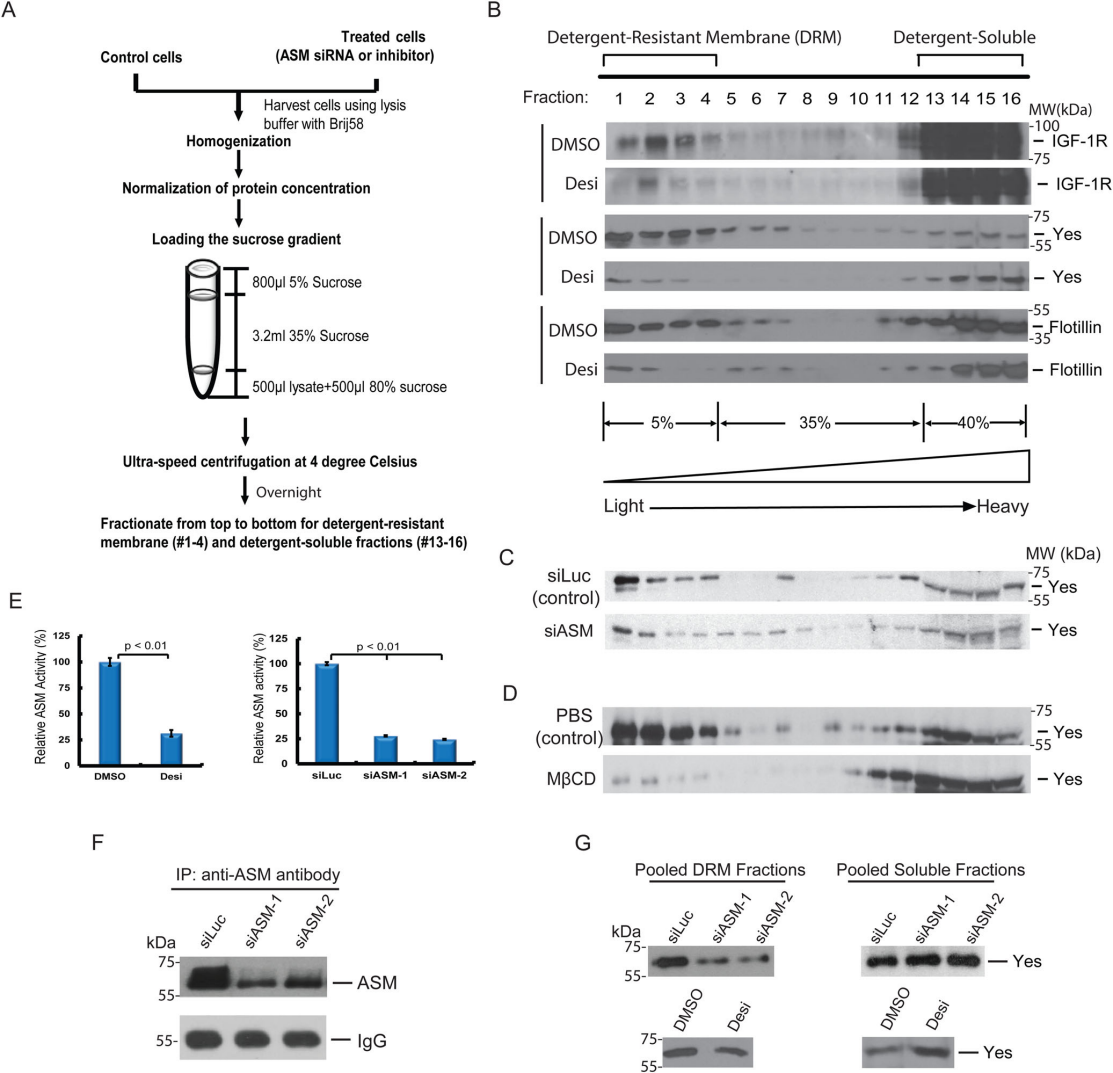
**Fractionation of the ASM-regulated membrane-associated proteins by discontinuous sucrose gradient ultracentrifugation.** (A) A schematic workflow of the discontinuous sucrose gradient fractionation procedure. (B) The distribution of tyrosine kinases IGF-1R and Yes in the discontinuous sucrose gradient in control (DMSO) and desipramine (Desi, 25 μM, 12 h) treated U373-MG cells by anti-IGF-1R and anti-Yes antibody immunoblotting. Flotillin was used as a lipid raft marker. Fractions were collected from the top (fraction #1) to the bottom of the gradient (fraction #16). The distribution of IGF-1R or Yes was reduced in the DRM fractions (#1–4) after ASM inhibition. (C) Loss of ASM reduced the levels of Yes in the detergent resistant membrane fraction. U373-MG cells were transfected with 50 nM control (luciferase, siLuc) and ASM siRNAs (siASM) for 48 h and the cells were harvested in the Brij58-containing lysis buffer and fractionated and analyzed as in B. (D) The same as in B except U373-MG cells were treated control (PBS) or 10 mM MβCD for 1 h and the cells were harvested in the Brij58-containing lysis buffer, fractionated and analyzed as in B. (E) The reduction of ASM activities in the desipramine-treated cells or the ASM siRNAs-treated cells were determined using the sphingomyelinase assay, as compared with control cells. Data are means±s.d. (*n*=3) with three independent repeats. (F) Knockdown efficiency of two independent ASM siRNAs on the ASM protein levels in U373-MG cells. ASM was immunoprecipitated from the lysates and then analyzed by western blot by an anti-ASM antibodies. (G) Independent verification of the effects of ASM siRNAs, desipramine and MβCD on the distribution of Yes in discontinuous sucrose gradient. U373-MG cells were transfected with 50 nM control (siLuc) and two independent ASM siRNAs for 48 h or treated with 25 μM desipramine for 10 h or 10 mM MβCD for 1 h. The cells were harvested in the Brij58-containing lysis buffer and fractionated. The distribution of Yes was monitored in the pooled DRM fractions (fraction #1–4) or soluble fractions (fraction #13–16) by western blotting.

The sphingomyelin-enriched lipid microdomains are known to be relatively resistant to nonionic detergents, such as Triton X-100, and can be isolated as ‘the detergent-resistant membrane (DRM) fractions’, which can be separated from the detergent-soluble fractions using a sucrose gradient and ultracentrifugation (Harder et al., 1998; Schuck et al., 2003). Since lipid microdomains are heterogeneous with varying lipid composition and protein content, their resistances to various detergents are known to be different (Giurisato et al., 2003; Radeva and Sharom, 2004; Schuck et al., 2003). The detergent Brij has been shown to preserve the lipid raft localization of transmembrane receptors (e.g. T cell receptor) better than Triton X-100 (Giurisato et al., 2003; Montixi et al., 1998; Röper et al., 2000). Human IGF-1R can also be fractionated in the detergent Brij-resistant membrane (DRM) fractions (Remacle-Bonnet et al., 2005). Since our genetic studies have established that the worm homolog of ASM regulates the IGF-1R-like signaling pathway in *C. elegans* (Kim and Sun, 2012), it is likely that human IGF-1R is also regulated by ASM. Indeed, in human glioblastoma U373-MG cells, which are highly sensitive to ASM inhibition (Zhu et al., 2016), we found there is a small fraction of IGF-1R localized in the DRM fractions (fraction #1–4). However, most of the IGF-1R protein was localized in the Brij-soluble fractions (fractions #13–16) (Fig. 1A,B). We also found that the detergent Brij58, rather than Triton X-100, was more efficient in preserving the lipid raft localization of IGF-1R (data not shown). In cells treated with desipramine, the localization of IGF-1R in the DRM fractions was reduced (Fig. 1B). Desipramine is a tricyclic amine anti-depression drug that acts as a functional inhibitor of ASM, and the drug blocks the interaction of ASM with membrane inside the lysosomes and causes ASM degradation (Albouz et al., 1981; Jaffrézou et al., 1995; Jenkins et al., 2011; Zhu et al., 2016). Indeed, the ASM activity was potently suppressed in cells treated with desipramine, confirmed by assaying the ASM activities using ^14^C-sphingomyelin as a substrate (Fig. 1E).

As control, we have found that Yes, a member of the src-family kinases known to be associated with lipid rafts (Radeva and Sharom, 2004; Schuck et al., 2003), is also localized in the Brij-DRM fractions (Fig. 1B). As the DRM fractions only contain 1% of total proteins (based on quantification of protein concentrations using the Bradford assay), our studies revealed that Yes is enriched in the DRM fractions. In cells treated with desipramine, there was a significantly reduced level of the Yes protein in the DRM fractions, while the protein level of Yes in the soluble fractions was slightly increased (Fig. 1B). In addition, we also found that a fraction of flotillin, a known lipid raft protein, also associated with the Brij58-resistant lipid membrane fractions and this membrane-associated fraction is also reduced after desipramine treatment (Fig. 1B). To confirm these results, we also used two different ASM siRNAs to verify the reduced levels of Yes protein in the DRM fractions in the ASM-inactivated cells (Fig. 1C and G). The Yes protein distribution in the pooled DRM fractions and pooled soluble fraction were also examined (Fig. 1G). In these experiments, the efficiency of ASM siRNAs on ASM gene silencing was confirmed by assaying the ASM activities using ^14^C-sphingomyelin as a substrate (Fig. 1E). The effects on ASM protein levels by ASM siRNAs were also confirmed by immunoprecipitation/western blot analysis, since ASM protein was too low in abundance to be detected by straight western blot analysis (Fig. 1F).

As cholesterol is reported to be involved in the formation of sphingomyelin-enriched lipid rafts, we also tested the effects of cholesterol depletion on membrane proteins. Methyl-β-cyclodextrin (MβCD) is a specific chemical that removes cholesterol from cultured cells. Previous reports showed that depletion of cellular cholesterol by MβCD is accompanied with the loss of sphingomyelin-enriched membrane rafts (Klein et al., 1995). We found that MβCD treatment also caused the reduction of Yes from the DRM fractions (Fig. 1D), suggesting that Yes is associated with lipid membranes that are sensitive to ASM and cholesterol depletion.

### Mass spectrometry analysis of ASM-dependent membrane-associated proteins

**Fig. 2.**
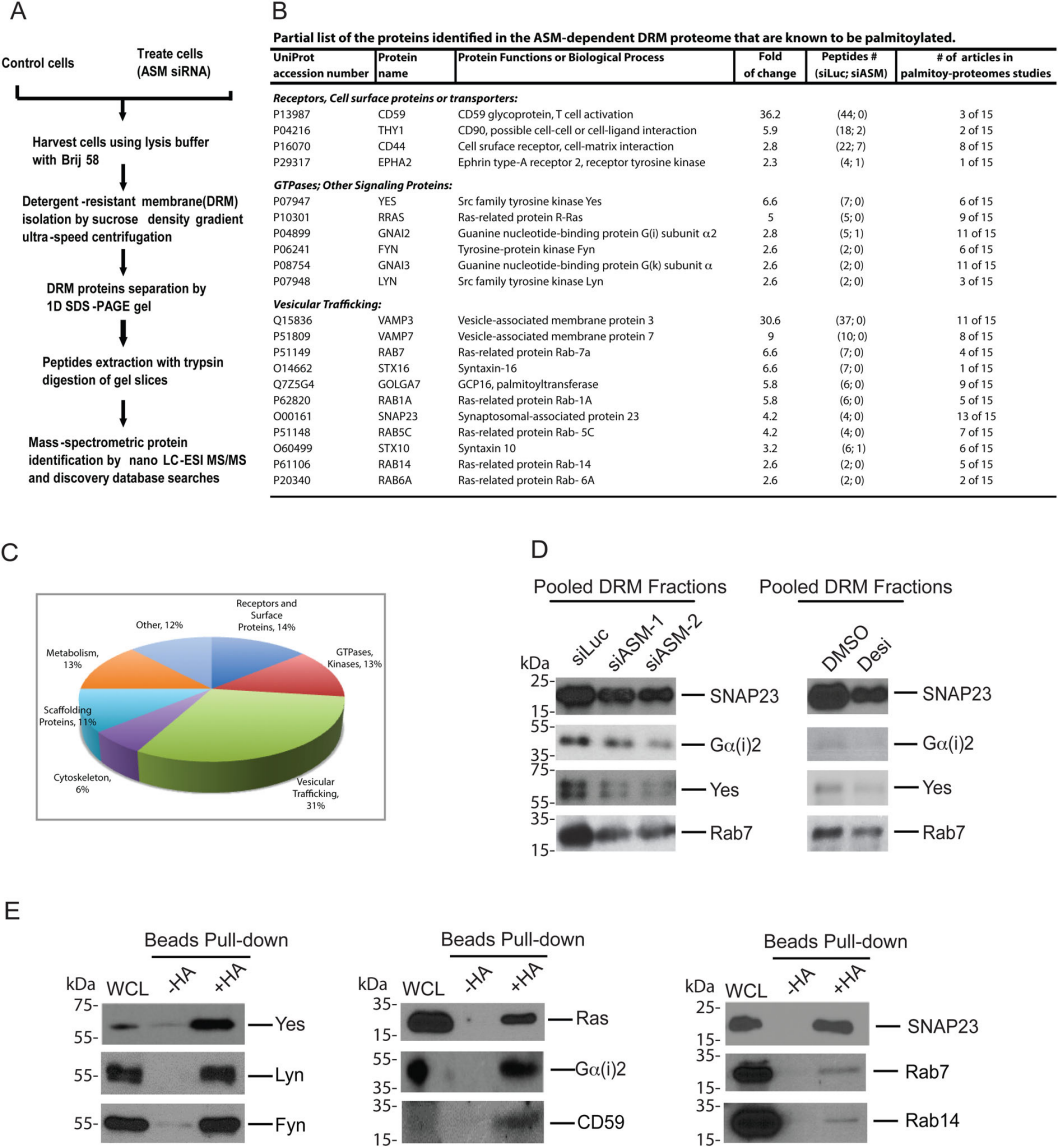
**Mass spectrometry-based proteomic analysis of ASM-regulated DRM-associated proteins.** (A) A schematic illustration of the proteomic procedure to analyze ASM-regulated DRM-associated proteins. (B) A partial list of proteins identified the ASM-sensitive DRM proteome. Proteins identified are cross-referenced to the palmitoyl-proteome database (SwissPalm) and 65 proteins are found to be known palmitoylated proteins (Table S3). Selected proteins from Table S3 are listed here. Semi-quantitative mass spectrometry data, fold of protein abundance change between control siLuciferase and siASM, as well as numbers of peptides identified in each condition, for the listed proteins are shown in the middle columns. The numbers of articles that reported the particular protein found in the palmitoyl-proteome is indicated in the last column. (C) A pie illustration to indicate the functional categories and the percentage of proteins in each category. Analysis is based on the identified palmitoylated proteins in the ASM-sensitive DRM proteome (Table S3). (D) Validation of the mass spectrometry-based quantitation by western blot analysis. Selected proteins from the list in panel B were subjected to western blot analysis using corresponding antibodies. Cells were treated with ASM siRNA or desipramine, or corresponding controls, and subjected by discontinuous sucrose gradient ultracentrifugation analysis. The pooled DRM fractions are analyzed as described in Fig. 1G. (E) The ABE assay to detect protein palmitoylation. Lysates from U373-MG cells were exposed to NEM to block all unmodified free thio groups (-SH) in the cysteine residues. The NEM-treated cell lysates were next exposed to hydroxylamine (HA), or left untreated. Both groups were then incubated with biotin-HPDP and subsequently pulled down by the streptavidin-agarose resins, followed by western blot analysis. Palmitoylated protein are enriched by HA treatment as compared to the no prior HA treatment group. Whole cell lysates (WCL) were used as control.

Having established a biochemical fractionation method to fractionate ASM-sensitive DRM proteins, we took a proteomic approach to interrogate the DRM-associated proteins that are potentially regulated by ASM (Fig. 2A). Briefly, U373-MG cells, treated with control or ASM specific siRNAs, were lysed in the Brij58-containing buffer and fractionated by ultracentrifugation on a sucrose gradient. The (DRM) fractions (fractions #1–4) were pooled, resolved on SDS protein gel, followed by protein identification using an ESI-LTQ-Orbitrap XL mass-spectrometer (Thermo Electron). Proteomic profiling data were first analyzed by QualBrowser in Xcalibur and Proteome Discoverer and then by the Scaffold software to identify proteins with unique peptides (described in detail in the Materials and Methods). The obtained data were then subjected to the label-free spectrum counting analysis, which has been used successfully to obtain a semi-quantitative difference of protein abundance in two sets of the samples (Liu et al., 2004; Old et al., 2005). Among 868 total protein hits analyzed, we have identified 108 DRM-associated proteins that show a greater than twofold reduction in the cells treated with ASM siRNAs (siASM), as compared with control cells treated with a non-specific luciferase siRNA (siLuc). These 108 proteins constituted a high confidence group, each identified by at least two unique peptides, in the ASM-sensitive DRM-associated proteome (Table S1). The next group are 64 proteins, each identified by at least one unique peptide, which show a change of abundance of >1.8-fold, and these 64 proteins constitute the low confidence group (Table S1). However, not all proteins identified have a reduced DRM association when ASM is knockdown. In fact, there are 116 of proteins that show a greater than twofold increase with the DRM association when ASM is inactivated, including a few members in the integrin family (Table S2). These observations suggest that there present a set of proteins which association with DRM requires the function of ASM.

### Enrichment of palmitoylated proteins in the ASM sensitive membrane fractions

When examining the list of proteins obtained by mass spectrometry analyses (Table S1), we noticed that the majority of the identified DRM proteins that are sensitive to the loss of ASM are peripherally membrane-associated proteins, whereas transmembrane proteins represent a relatively small group. This could be due to a poor recovery of the transmembrane proteins by the isolation procedure or due to the low abundance of the transmembrane proteins present in the DRM fractions. We focused on characterizing the peripherally membrane-associated proteins to investigate how these proteins might be associated with the ceramide-rich lipid rafts, as inactivation of ASM should reduce the levels of ceramides in the membrane. We wondered whether these proteins contain any common structural elements that help confer their membrane lipid association properties.

During these mass spectrometry-based proteomic analyses, we again recovered Yes as one of the ASM-sensitive DRM proteins (Fig. 2B,D). Yes is known to be tethered to the cytosolic face (the inner leaflet) of plasma membrane by palmitoylation (Resh, 1994; Sandilands et al., 2007). Since palmitoylation is the most common acylation event in eukaryotes that allows otherwise cytosolic proteins to be attached to the plasma membrane, we wondered if our ASM-sensitive proteome has a selective enrichment of proteins that are palmitoylated. We therefore closely examined whether there are additional palmitoylated proteins in our top list of the protein hits through database search of reported literature and also by cross-referencing the protein palmitoylation status in a palmitoyl-proteome database, the SwissPalm (Blanc et al., 2015). Indeed, among 108 proteins in our high-confidence group of the ASM-sensitive proteome, we have found that 60% of these proteins (65 out of 108 proteins) are known to be palmitoylated (Table S3), according to the previous studies by palmitoyl-proteomics analyses and other studies (Kang et al., 2008; Rocks et al., 2010; Serwa et al., 2015). These 65 proteins can be categorized into cell surface receptors (14%), SFKs and Ras-family GTPases involved in cell signaling (13%), proteins participated in vesicular trafficking (31%), cytoskeleton reorganization (6%), scaffold proteins (11%), metabolism (13%) and other functions (12%) (Fig. 2C). Some examples of the palmitoylated proteins discovered in our ASM-sensitive DRM proteome are listed in Fig. 2B.

To verify the results from the mass spectrometry-based proteomic analyses, we have examined several representative proteins by independent western blot analysis. Our analysis revealed that similar to Yes, the levels of SNAP23 (the synaptosome-associated protein of 23 kDa), Gα(i)2 (GNAI2, heterotrimeric G protein subunit) and Rab-family of GTPases Rab7, were reduced in the DRM fractions after cells were treated with ASM siRNAs (Fig. 2D). The membrane association of these proteins was also sensitive to ASM inhibitor desipramine (Fig. 2D). These studies indicate that our semi-quantitative mass-spectrometry analysis provides an effective way to identify the ASM-sensitive, DRM-associated proteins.

### The ASM-sensitive and DRM-localized proteins are palmitoylated proteins

Our proteomic approach has identified proteins such as SNAP23, Yes and Gα(i)2 that were previously reported to be palmitoylated in other cells under various conditions. However, since palmitoylation is a dynamic post-translational process, we would like to ensure that these proteins are palmitoylated under our culture conditions in U373-MG cells. We therefore have used the Acyl-Biotinyl Exchange (ABE) assay to examine the palmitoylation status of various proteins we have identified (Fig. 2B and E). In the ABE assay (Kang et al., 2008; Roth et al., 2006), sequential chemical modifications were used to selectively label the palmitoylated proteins by biotin-HPDP, a sulfhydryl-reactive biotinylation reagent. The biotin-HPDP labeled proteins (palmitoylated proteins) can be pulled down by the streptavidin-agarose resins. Using this specific assay, we found that Yes, Lyn, Fyn, Ras, Gα(i)2, CD59, SNAP23, Rab7 and Rab14, which were identified in our ASM-sensitive DRM proteome (Fig. 2B), were all palmitoylated proteins in U373-MG cells (Fig. 2E). Therefore, our proteome analysis selectively identified a large number of endogenous palmitoylated proteins that are enriched in ASM-regulated DRM fractions in U373-MG cells.

### Loss of ASM diminishes the plasma membrane distribution of palmitoylated proteins

**Fig. 3.**
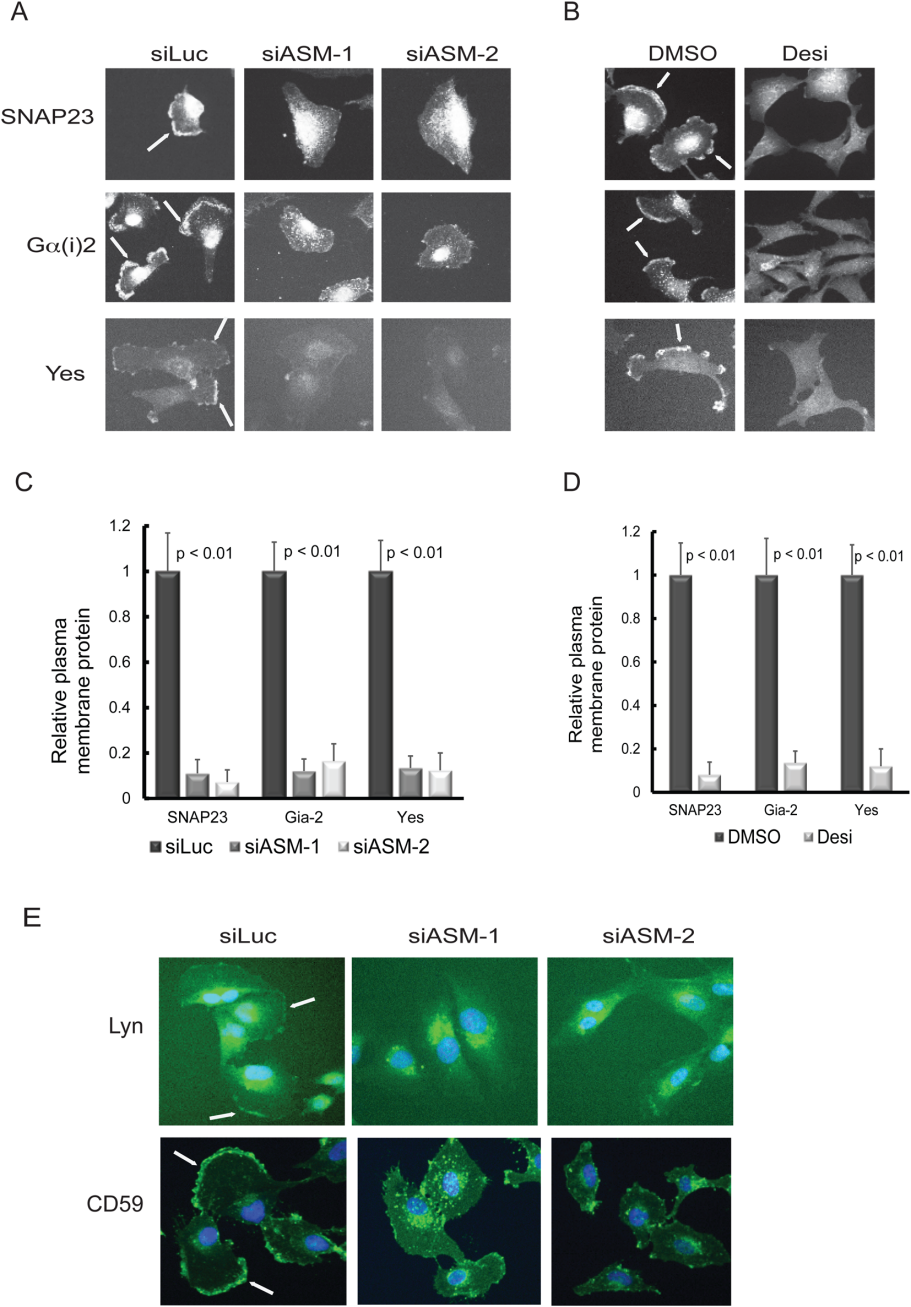
**ASM-deficiency reduces the plasma membrane localization of the palmitoylated proteins.** (A,B) Selective inactivation of ASM by two independent siRNA (A) or ASM inhibitor desipramine (B) reduced the plasma membrane localization of SNAP23, Gα(i)2 and Yes, as compared with cells treated with corresponding controls. Cells were processed by immunostaining with specific antibodies and examined by fluorescence microscopy. The plasma membrane staining of specific proteins is indicated by arrows. (C,D) Quantification of cell imaging data from A and B. Inhibition of ASM leads to strong reduction of SNAP23, Gα(i)2 and Yes on cell membranes. Relative plasma membrane staining was obtained by quantitation of the intensity of the fluorescence staining on the plasma membrane as compared with the intensity of the staining in the whole cells (200 cells were measured for each condition), using ImageJ software. Experiments were repeated three times to derive the mean of the relative abundance for the plasma membrane associated protein (statistically significant, *P*<0.01, Student's *t*-test). (E) Silencing ASM also reduces the plasma membrane staining of Lyn and CD59. Cells were treated with two independent ASM siRNAs and stained with antibodies for Lyn, or CD59, respectively (green), and counter stained with DAPI, a DNA binding dye to stain the nucleus (blue). Scale bar: 10 μm.

Recent studies show that the Golgi apparatus is a major organelle to carry out the palmitoylation of proteins, e.g. H-Ras and Fyn, which undergo dynamic cycling between the Golgi and plasma membrane (Rocks et al., 2010, 2005). These proteins are found to be palmitoylated on the Golgi, trafficked to the plasma membrane, and then rapidly depalmitoylated and returned to the Golgi, with a cycling time of about 20–30 min. We wondered whether ASM is involved in the regulation of the rapid subcellular cycling of the palmitoylated proteins. We therefore examined the cellular localization of a group of palmitoylated proteins, including SNAP23, Yes, and Gα(i)2, by immunostaining. Our examination revealed that these endogenous proteins all showed a prominent localization in the plasma membrane in the control U373-MG cells (Fig. 3A,B, indicated by arrows). However, we found that knockdown of ASM by two independent siRNAs or treatment with desipramine both greatly abolished the plasma membrane localization of these proteins (Fig. 3A,B). Our quantification of these analyses indicated that inactivation of ASM greatly diminished the plasma membrane distribution of these proteins (Fig. 3C,D). We also further examined the effects of ASM inactivation on other palmitoylated proteins such as Lyn and CD59. Our studies revealed that while both Lyn and CD59 are present on plasma membrane in control cells, siRNA-mediated knockdown of ASM led to the disappearance of these proteins from the plasma membrane (Fig. 3E). Our cell-based distribution studies, together with our mass spectrometry-based proteomic profiling, indicate that the palmitoylated proteins are enriched in ASM-sensitive DRM fractions and that ASM is required for the localization of these palmitoylated proteins to the plasma membrane.

### ASM deficiency causes the accumulation of SNAP23 in the trans-Golgi network (TGN)

**Fig. 4.**
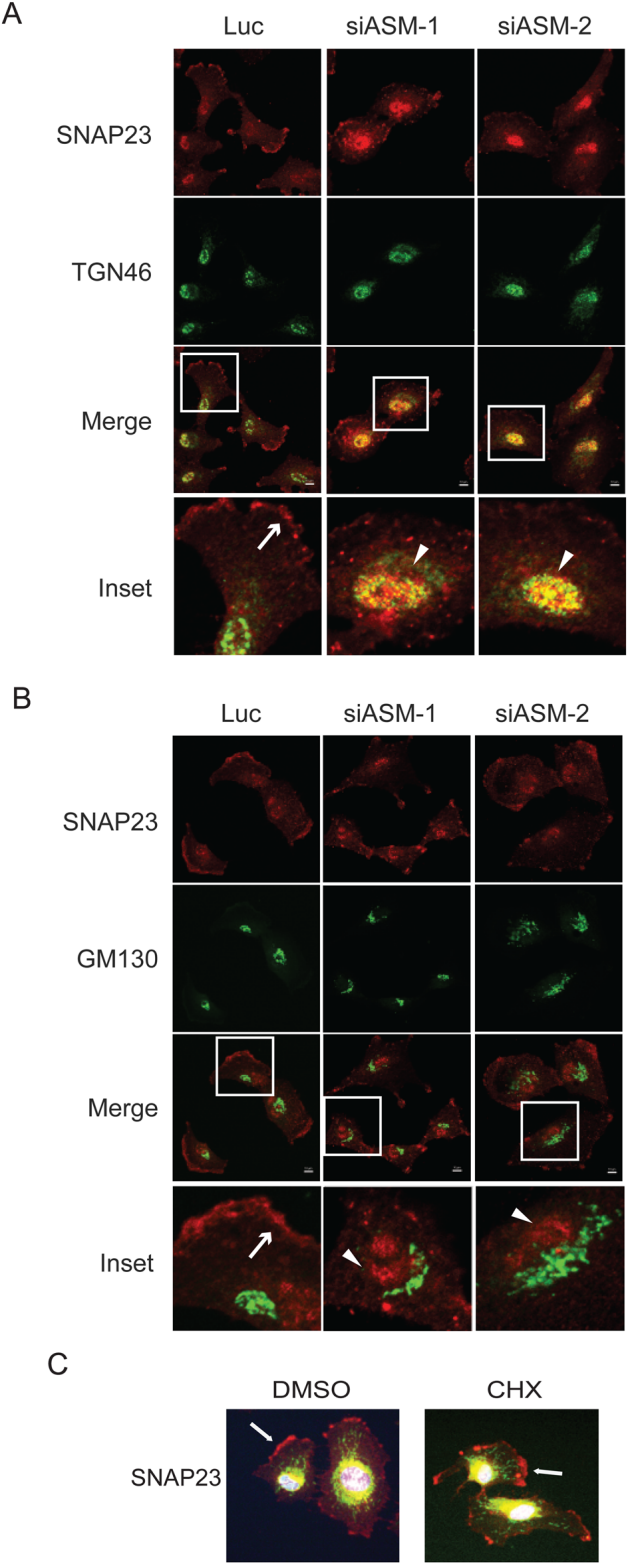
**Inactivation of ASM blocks the plasma membrane delivery of SNAP23 with concurrent accumulation in TGN.** (A,B) U373-MG cells were transfected with 50 nM control (Luc) or two independent ASM siRNAs for 48 h. Cells were co-immunostained with anti-SNAP23 (red) and TGN marker TGN46 (green) in panel A, or SNAP23 (red) and cis-Golgi marker GM130 (green) in panel B. Inactivation of ASM leads SNAP23 to disappear from the plasma membrane (indicated by arrows) and accumulated intracellularly (indicated by arrowheads) that are co-localized with TGN46 (yellow) but not with GM130. Inset: boxed regions are enlarged. (C) U373-MG cells were treated with cycloheximide (50 μg/ml, CHX), or vehicle DMSO for 6 h and stained as in panel A. Scale bar: 10 µm.

We have noticed that while loss of ASM causes the disappearance of these proteins on the plasma membrane, some proteins, such as SNAP23, started to accumulate in the intracellular perinuclear region (Fig. 3A,B). SNAP23 is a SNARE protein that is ubiquitously expressed in a variety of cells and is involved in a wide array of diverse vesicle membrane–membrane fusion events including exocytosis from mast cells, insulin-dependent release of GLUT4 from adipocytes and neuronal postsynaptic glutamate receptor trafficking (Prescott et al., 2009). SNAP23 contains a centrally localized cluster of five cysteine residues that are shown to be linked to palmitoyl chains (Salaun et al., 2005) and our studies showed that SNAP23 is indeed palmitoylated in U373-MG cells (Fig. 2E). To determine in which cellular compartment that SNAP23 may be trapped by the ASM deficiency in U373-MG cells, we conducted co-immunostaining experiments. Our examination revealed that siRNA-mediated knockdown of ASM caused the accumulation of SNAP23 in the TGN region, colocalized with the TGN marker TGN46, but not with the cis-Golgi marker GM130 (Fig. 4A,B). We also examined the possibility that ASM inactivation may block the newly synthesized SNAP23 to be trafficked to the plasma membrane through the TGN (Fig. 4C). However, treatment of cells with protein synthesis inhibitor, cycloheximide, did not block the presence of SNAP23 on the plasma membrane (Fig. 4C), suggesting SNAP23 is being recycled through the TGN to appear on the plasma membrane. Our results indicate that ASM inactivation caused a failure of transport of SNAP23 out of the TGN.

### Inhibition of palmitoylation prevents the plasma membrane localization of SNAP23

**Fig. 5.**
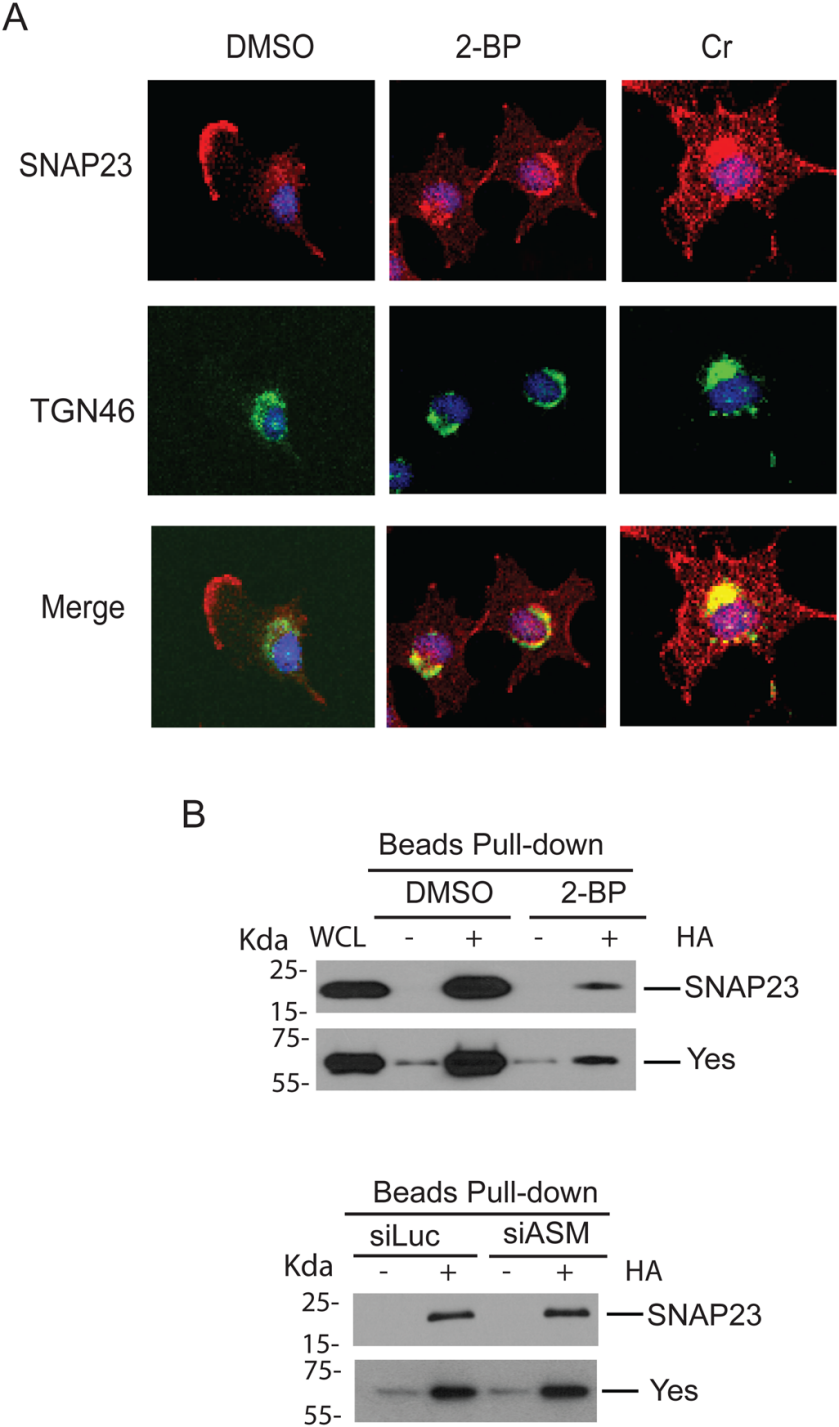
**Palmitoylation regulates the plasma membrane delivery and membrane-association of palmitoylated proteins.** (A) Palmitoylation inhibitors block the appearance of SNAP23 on the plasma membrane and cause SNAP23 accumulation in the TGN. U373-MG cells were treated with palmitoylation inhibitors 2-bromopalmitate (2-BP, 100 µM) or cerulenin (Cr, 5 µg/ml) for 6 h. Cells were co-immunostained for SNAP23 (red) and the TGN marker TGN46 (green), and counter-stained with DAPI for nuclei (blue). Both drug treatment lead to a disappearance of SNAP23 on the plasma membrane and the co-localization of SNAP23 with TGN46 (yellow), indicating trapping of SNAP23 in the TGN. (B) Inactivation of ASM does not affect protein palmitoylation. Palmitoylation of SNAP23 and Yes was each greatly reduced after treatment of cells with 2-bromopalmitate (upper panels) but not after ASM knockdown. U373-MG cells were treated with 100 µM 2-bromopalmitate (2-BP) for 6 h or transfected with 50 nM control and ASM siRNAs for 48 h. Palmitoylation of SNAP23 and Yes were analyzed using the ABE assay, as in Fig. 2E.

Since our studies indicate that ASM is required for the distribution of palmitoylated proteins such as SNAP23 to the plasma membrane (Figs 3, 4), we tried to determine whether palmitoylation is indeed required for SNAP23 to appear on the plasma membrane in our system. We used two types of palmitoylation inhibitors, 2-bromopalmitate (2-BP) or cerulenin (Cr) (Resh, 2006), to block the palmitoylation of SNAP23 and examined the changes of SNAP23 localization on the plasma membrane. Our studies showed that treatment of cells with 2-bromopalmitate or cerulenin greatly reduced the plasma membrane-associated SNAP23 (Fig. 5A). Using the ABE assay, we found that the levels of palmitoylated SNAP23 proteins were significantly inhibited by these palmitoylation inhibitors (Fig. 5B). Similarly, these palmitoylation inhibitors also markedly diminished the palmitoylation levels of Yes (Fig. 5B). These studies again confirmed that SNAP23 is indeed palmitoylated in our cells, and the palmitoylation status affects SNAP23 intracellular localization. Notably, our studies revealed that SNAP23 staining on the plasma membrane disappeared after the treatment of these palmitoylation inhibitors with concurrent accumulation in the TGN region that overlapped with the TGN46 marker (Fig. 5A), indicating that SNAP23 is palmitoylated in the TGN and its trafficking out of the TGN requires a prior palmitoylation of the protein. Although there is a study suggesting that the palmitoylation of SNAP23 occurs on the plasma membrane (Weber et al., 2017), our data are consistent with the report showing that the SNAP23 palmitoylation occurs on Golgi (Salaun et al., 2005). Since palmitoylation inhibitors phenocopied the effects of ASM inactivation (comparing Figs 4A and 5A) on the dynamic distribution of SNAP23 from the plasma membrane to the TGN, our studies strongly suggest that ASM regulates the palmitoylation-dependent vesicle transport of SNAP23 from the TGN to the plasma membrane.

### ASM does not affect protein palmitoylation process itself

Since treatment of cells with palmitoylation inhibitors phenocopied the effects of ASM inactivation on the distribution of SNAP23 on the plasma membrane and the TGN (Fig. 5A), it remains unclear whether loss of ASM affects the palmitoylation process itself. To determine whether ASM acts at the palmitoylation step or after palmitoylation of SNAP23, we directly tested whether loss of ASM affects palmitoylation of SNAP23 or other proteins using the ABE assay. We found that while treatment of cells with 2-bromopalmitate greatly inhibited the palmitoylation of SNAP23 and Yes, however, knocking down of ASM did not affect the palmitoylation levels of these proteins (Fig. 5B). Our studies thus indicate that ASM is not required for the palmitoylation reaction of SNAP23 or other proteins per se. Rather, ASM inactivation is likely to affect the association of SNAP23 and other palmitoylated proteins with certain membrane components in the TGN, causing the failure of these proteins to be transported out of the TGN.

### Palmitoylation mutants fail to partition in the ASM-regulated membrane fractions

**Fig. 6.**
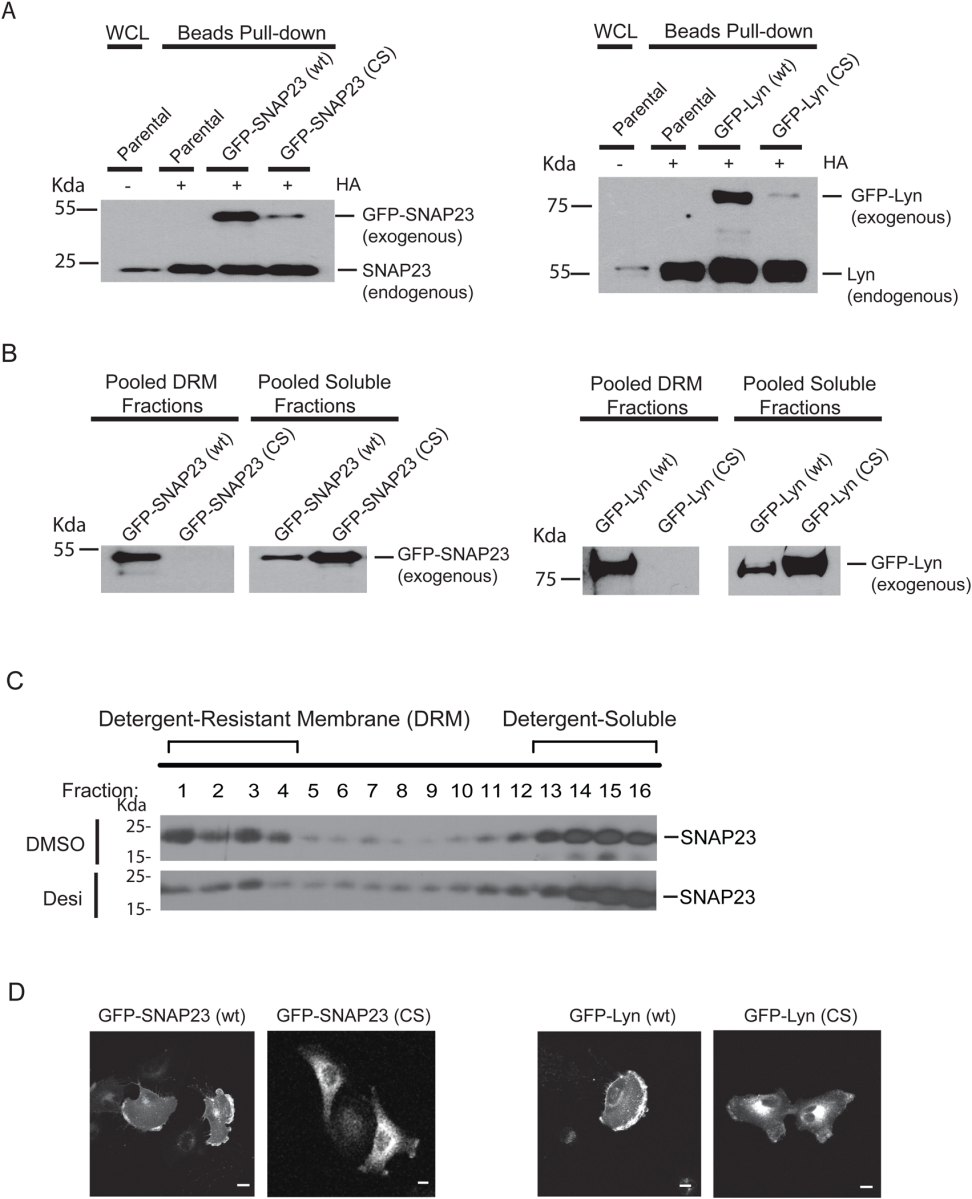
**DRM-association and intracellular localization of the palmitoylation-defective SNAP23 and Lyn.** (A) Mutations of specific cysteines in SNAP23 and Lyn greatly reduced their palmitoylation. Wild-type (wt) and cysteine to serine (CS) mutants of SNAP23 and LYN were expressed as GFP-SNAP23 and GFP-Lyn fusion proteins, with GFP tagged at the N-terminus of SNAP23 and C-terminus of Lyn, respectively. Expression constructs were stably transfected into U373-MG cells. The cell lysates were prepared and palmitoylation of the wild type and CS mutants were analyzed using the ABE assay as in Fig. 2E. While the exogenous CS mutant of SNAP23 and Lyn has greatly reduced palmitoylation levels, the palmitoylation status of the endogenous SNAP23 and Lyn are not affected, further serving as controls. (B) Cysteine mutants of SNAP23 and Lyn failed to associate with the DRM fractions. Wild-type and CS mutants of GFP-SNAP23 and GFP-Lyn proteins were stably transfected into U373-MG cells. The cells were lysed in Brij58-containing lysis buffer and fractionated in the discontinuous sucrose gradient ultracentrifugation, as in Fig. 1B, and the pooled fractions were examined by western blot analysis, as described in Fig. 1G. Only the wild-type but not the CS mutant proteins are found in the DRM fractions, while the CS mutant proteins are found mostly in the pooled soluble fractions. (C) The association of SNAP23 with the DRM fractions is sensitive to the ASM inhibitor desipramine. U373-MG cells were treated with the ASM inhibitor desipramine or vehicle control DMSO for 12 h. Lysates were prepared in the Brij58 lysis buffer and subjected to the sucrose discontinuous sucrose gradient ultracentrifugation as described in Fig. 1B. The fractions were analyzed by western blot analysis using anti-SNAP23 antibodies. (D) CS mutants of GFP-SNAP23 and GFP-Lyn failed to appear on the plasma membrane and were accumulated in the intracellular compartment. Wild-type and CS mutants of GFP-SNAP23 and GFP-Lyn constructs were transfected into U373-MG cells. The localization of the GFP-tagged ectopically expressed proteins on the plasma membrane and intracellular compartment was examined by fluorescence microscopy. Scale bars: 10 µm.

SNAP23 contains a centrally localized cluster of five cysteine residues, C79, C80, C83, C85 and C87 that are known be palmitoylated (Salaun et al., 2005). These five cysteine residues are also predicted to be palmitoylated according to the SwissPalm database analysis (Blanc et al., 2015). To further characterize the role of palmitoylation in SNAP23 in association with the ASM-sensitive DRM fractions, we have converted these cysteine residues to serine residues (C79S, C80S, C83S, C85S and C87S), and the C->S mutant derivative should not be palmitoylated. We also mutated the cysteine residues at position 3 and 381 in Lyn to serines (C3S, C381S), since the cysteine 3 residue is known to be palmitoylated (Kovarova et al., 2001), and both cysteine 3 and 381 are predicted to be palmitoylated according to the SwissPalm database analysis (Blanc et al., 2015). To determine the role of palmitoylation in regulation of DRM association, we examined and compared the behavioral differences of the Cys->Ser mutants of SNAP23 and Lyn to their wild-type counterparts after ectopically expressing them in cells. To distinguish the ectopically expressed proteins from the endogenous ones, we have expressed the ectopically expressed proteins as GFP fusion proteins, with GFP tagged at the N-terminus of SNAP23 and the C-terminus of Lyn, respectively (Fig. 6A,B). Expression of these proteins in U373-MG cells revealed that the Cys->Ser mutations in SNAP23 and Lyn greatly reduced their respective palmitoylation levels based on the ABE assay, as compared to their wild-type counterparts (Fig. 6A). In addition, while the exogenous GFP-SNAP23(CS) or GFP-Lyn(CS) mutant proteins failed to be palmitoylated, the palmitoylation of the endogenous SNAP23 and Lyn proteins are not affected, serving as a positive control for each cell line (Fig. 6A). In addition, we found that these palmitoylation defective mutants of SNAP23 and Lyn also failed to associate with the DRM fractions as analyzed by the discontinuous sucrose gradient ultracentrifugation method (Fig. 6B). In comparison, the distribution of the endogenous SNAP23 in these DRM fractions was sensitive to the ASM inhibitor desipramine (Fig. 6C), in a manner similar to the behavior of Yes (Fig. 1B). These studies showed that the palmitoylation of SNAP23 and Lyn is required for their association with the ASM-regulated DRM compartments. By fluorescence microscopy, our studies revealed that while the wild-type GFP-SNAP23 and GFP-Lyn proteins were both found on the plasma membrane, their palmitoylation-defective mutants failed to appear on the plasma membrane (Fig. 6D). In fact, we found that these mutant proteins accumulated intracellularly, likely in the Golgi area (Fig. 6D). Here again, our studies indicate that the behavior of these palmitoylation-defective mutants of SNAP23 and Lyn phenocopied that of their endogenous proteins in response to the ASM inactivation (Figs 4, 6). Thus, our studies provide strong evidence that palmitoylation of these proteins is responsible for their association with the ASM-sensitive DRM fractions, which correlates with their ability to be localized on the plasma membrane, likely through trafficking out of the TGN.

### Both ASM and ceramides are present on the cell surface

**Fig. 7.**
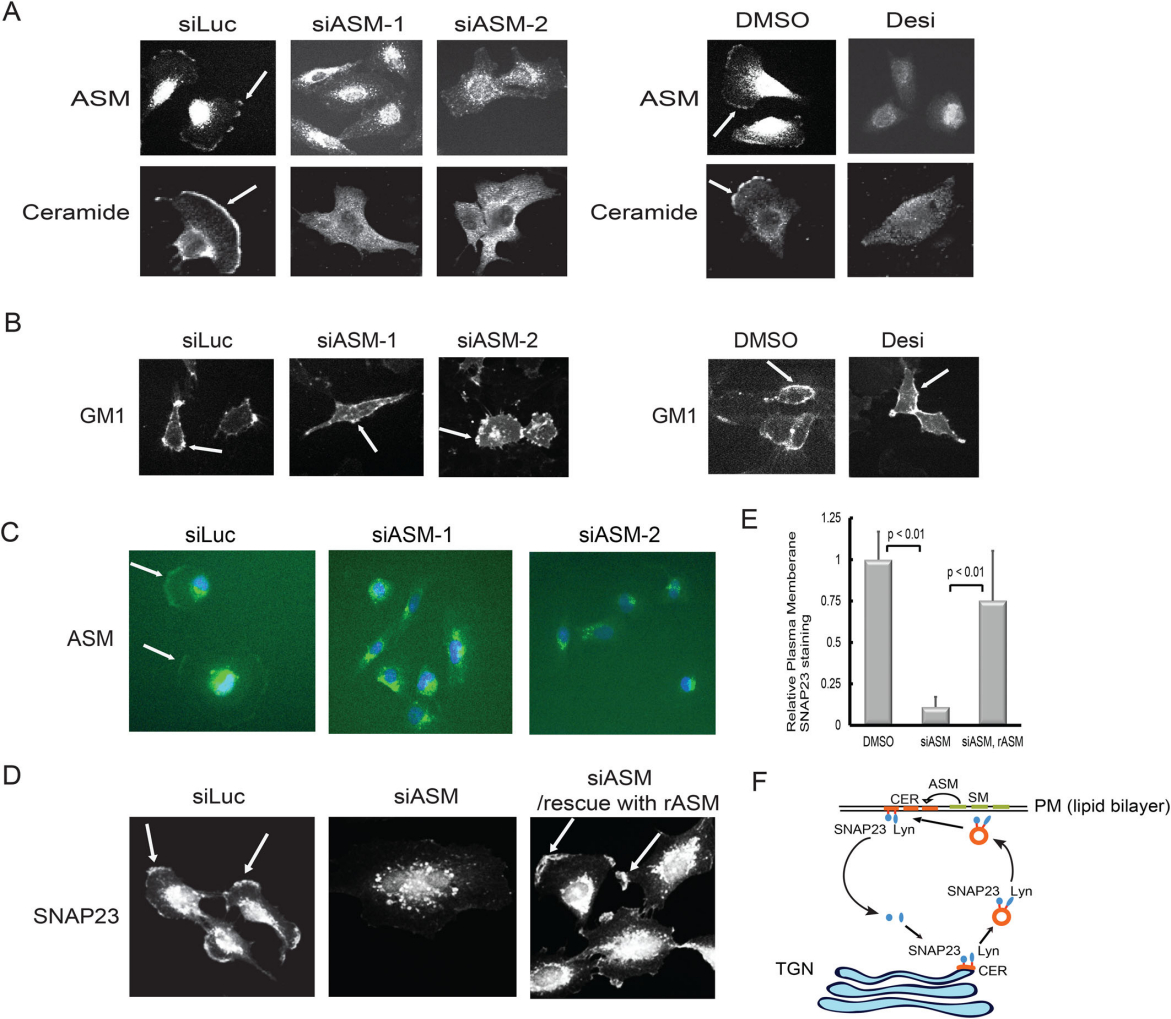
**ASM acts at the plasma membrane to regulate the intracellular trafficking of palmitoylated proteins.** (A) U373-MG cells were treated with 50 nM control and two independent ASM siRNAs for 48 h. Alternatively, cells were treated with 25 μM desipramine or DMSO for 12 h. The localization of ASM and its catalytic product, ceramide, was examined by specific anti-ASM and anti-ceramide antibody immunostaining. The plasma membrane staining of both ASM and ceramide (indicated by arrows) disappeared in cells after treatment with ASM siRNAs or with the ASM inhibitor desipramine. (B) The distribution of ganglioside GM1 on the plasma membrane is not affected by ASM inactivation. The experiments were conducted similarly as panel A except that cells were immunostained with an anti-GM1 antibody (immunostaining of GM1 on the plasma membrane is indicated by arrows). (C) Cells were transfected with siRNAs as in panel A for 48 h. Live cells were stained directly with anti-ASM antibodies at room temperature for 1 h. Cells were then fixed and processed for staining with by secondary antibodies and imaging. (D) Extracellular recombinant ASM enzyme can rescue the trafficking defects of SNAP23 in the ASM siRNA-treated cells. U373-MG cells were transfected with 50 nM control and ASM siRNAs for 48 h. In one set of the ASM siRNA-treated cells (right panel), purified recombinant active recombinant human ASM enzyme (4 μg/ml final) was added to the culture media for 2 h. Cells were then fixed, processed by immunostaining with anti-SNAP23 antibody, and examined by fluorescence microscopy. (E) For quantification the relative abundance of SNAP23 under all three conditions in panel C was measured (200 cells from each condition), as in Fig. 3C, and then normalized to control cells. Three independent experiments were measured to obtain the average of SNAP23 distribution on the plasma membrane (statistically significant, *P*<0.01, Student's *t*-test). (F) A model for the ASM function in regulation of SNAP23 and Lyn trafficking. ASM acts at the outer-leaflet of the plasma membrane (PM) to convert sphingomyelins (SM) to ceramides (CER). While CER are found in both leaflets of the plasma membrane, SM are mainly on the outer leaflet of the plasma membrane. Ceramides can retrogradely traffic back to Golgi (TGN). On the Golgi membrane, CER recruit the palmitoylated proteins such as SNAP23 and Lyn to facilitate the transport of these proteins out of the Golgi. SNAP23 and Lyn are carried by the ceramide-rich transport vesicles (possibly endosomes) en route to the plasma membrane, which eventually fused with the plasma membrane. On the plasma membrane, SNAP23 and Lyn are also associated with CER-rich lipid microdomains. When SNAP23 and Lyn are depalmitoylated, the depalmitoylated proteins become cytosolic and then associate with Golgi to be palmitoylated again, starting another cycle of trafficking. When ASM is inactivated, SNAP23 and Lyn are trapped in the Golgi, due to the lack of the CER on the Golgi membrane, which are essential for the transport of the palmitoylated proteins out of the Golgi. Scale bar: 10 μm.

We wondered if ASM might be localized on the plasma membrane, a location to allow an easy access to its substrates, sphingomyelins, which are asymmetrically localized on the outer-leaflet of the plasma membrane (Lopez-Montero et al., 2010; van Blitterswijk et al., 2003). We used specific anti-ASM antibodies to examine the cellular location of ASM. Our studies revealed that the ASM protein is indeed found on the plasma membrane (Fig. 7A). The immunostaining of ASM on the plasma membrane was specific since this staining was abolished in cells treated with ASM siRNAs or ASM inhibitor desipramine (Fig. 7A). Earlier studies have shown that when cells are exposed to various lethal doses of stress stimuli, the ASM protein is translocated from intracellular store(s) to the outer leaflet of the plasma membrane (Cremesti et al., 2001; Grassmé et al., 2003; Tam et al., 2010). Our data, however, indicate that ASM is normally present on the plasma membrane in actively growing U373-MG cells even without the presence of stress stimuli. To further determine that ASM protein is present on the plasma membrane, we immunostained live cells directly using anti-ASM antibody without prior fixation. Indeed, plasma membrane ASM staining was present in the control cells but not in the cells pretreated with ASM siRNAs (Fig. 7C). Under this staining condition, there is some staining in the perinuclear regions in both control cells and cells treated with ASM siRNAs, which might have occurred due to the antibodies being endocytosed during the live staining process (Fig. 7C).

We also used a specific monoclonal anti-ceramide antibody to determine whether ceramides, the products from the action of ASM on sphingomyelins, are found on the plasma membrane. Our immunostaining of ceramides revealed that ceramides are indeed present on the plasma membrane and such ceramide-immunostaining on the cell membrane surface was abolished in cells treated with either ASM siRNAs or ASM inhibitor desipramine (Fig. 7A). The effects of ASM on the plasma membrane lipids were selective, as we did not observe significant staining changes of GM1, a member of gangliosides, on the plasma membrane in cells treated with ASM siRNAs or ASM inhibitor desipramine (Fig. 7B). Our detection of ceramides in the plasma membrane is consistent with earlier reports that ceramides are detected as a patch region on the plasma membrane, although those ceramide-stained areas were only found on cells when challenged with strong stress stimuli (Cremesti et al., 2001; Grassmé et al., 2003; Tam et al., 2010).

### Rescuing the ASM siRNA-mediated trafficking block of SNAP23 by the extracellular ASM enzyme

Since ASM and ceramides are detected on the plasma membrane (Fig. 7A–C), we wondered whether the extracellularly localized ASM could indeed act through ceramides on the plasma membrane to regulate the intracellular trafficking of palmitoylated proteins from the Golgi to the plasma membrane. To test this possibility, we performed a ‘rescue’ experiment to determine whether an exogenously supplied recombinant ASM enzyme can rescue the SNAP23 trafficking defect in the ASM-deficient cells. For this experiment, we first silenced the ASM gene expression by specific ASM siRNAs for 48 h, and then incubated these ASM-deficient cells with the active recombinant human ASM protein (rASM), or left untreated (control). As shown in Fig. 7D, treatment of cells with ASM siRNAs, but not the control siRNAs, caused the disappearance of the plasma membrane staining of SNAP23. However, externally addition of the recombinant ASM protein to the ASM siRNA-treated cells was sufficient to allow the re-appearance of SNAP23 on the plasma membrane (Fig. 7D, and quantification in 7E). These results demonstrate that the presence of ASM enzyme outside of the cell is able to reverse the phenotypes of trapping SNAP23 in the TGN in the ASM-deficient cells, allowing SNAP23 to exit the TGN and then to traffic to the plasma membrane. These experiments suggest that ASM protein may function extracellularly to produce ceramides by hydrolyzing sphingomyelins on the outer leaflet on the plasma membrane to regulate the intracellular trafficking of the Golgi-localized palmitoylated proteins.

## DISCUSSION

In this report, we present our studies on using semi-quantitative mass-spectrometry analyses to interrogate the proteins that are associated with the DRM fractions. While more than 100 ASM-sensitive DRM-associated proteins were identified in the actively growing U373-MG cells, our investigation revealed that a major fraction (60%) of these proteins are palmitoylated proteins, including SFKs, Ras-family GTPases, Rab-family GTPases and proteins involved in vesicular trafficking such as SNAP23 and syntaxins (Fig. 2B; Table S3). Our results indicate that ASM and its catalytic products, ceramides, are required for the association of these palmitoylated proteins to the DRM fractions, both at the plasma membrane and at the Golgi membranes or endosome membranes. Our characterization of the palmitoylation-defective mutants of SNAP23 and Lyn suggests that the palmitoyl moiety in these proteins confers their association to the ASM-sensitive DRM fractions.

Our studies have also revealed a critical role of ASM in regulation of the trafficking of palmitoylated proteins. We found that inactivation of ASM causes a number of proteins, including SNAP23, Lyn, Yes, Gα(i)2 and CD59, to disappear from the plasma membrane, and concurrently promotes the accumulation of these proteins intracellularly (Fig. 3). Using SNAP23 as an example, we showed that ASM deficiency leads to SNAP23 accumulation at the Golgi, which can be phenocopied by treating cells with palmitoylation inhibitors or by mutation of the palmitoylation sites in SNAP23 (Figs 4–6). Similar effect was observed with Lyn (Fig. 6). While it is possible that ASM deficiency causes a defect in palmitoylation of SNAP23 and Lyn to account for their Golgi transport defects, our subsequent characterization showed that ASM did not affect the palmitoylation itself. Instead, ASM inactivation leads to a trafficking defect of these proteins to exit out of the TGN after they are palmitoylated. Interestingly, we have recently found that ASM deficiency blocked the trafficking of the MET receptor protein tyrosine kinase out of the Golgi to the plasma membrane, while the transport of a viral protein VSVG was not affected by ASM inactivation (Zhu et al., 2016). Therefore, it is possible that ASM is required for the selective transport of certain cargos from specific subdomains in the Golgi. In fact, the lipid microdomains of sphingomyelins and cholesterols have been proposed to act as a sorting platform on the Golgi to help proteins transport (Schuck and Simons, 2004). Our studies have added one additional layer of complexity to this model. It can be envisioned that ceramides on the Golgi membrane can provide a favorable lipid microenvironment to allow the palmitoylated proteins to interact with the Golgi transport machinery, although this case is to facilitate the transport carrier formation, rather than sorting per se. It is worth pointing out that our ASM-dependent DRM proteome analysis has identified many ASM-regulated proteins that are involved in various processes of vesicular transport, such as SNARE proteins and members of Rab GTPases. Future studies are required to unravel the specific roles of ceramides in the regulation of Golgi transport.

Our studies have shown that ASM protein and ceramides are present on the plasma membrane in the actively growing cells (Fig. 7). This is both consistent but different from the previous reports, which showed that lethal doses of stress stimuli could stimulate the transport of ASM from the intracellular stores, likely the luminal side of the lysosomes, to the extracytoplasmic side of the plasma membrane (Cremesti et al., 2001; Grassmé et al., 2003; Tam et al., 2010). Specifically, we found that high levels of ASM proteins and ceramides are present on the plasma membrane in actively growing glioblastoma cells, even without exposure to stress stimuli. The presence of ASM and ceramides on the plasma membrane of actively growing cells suggests that the enzyme and the lipids have a normal function in cells, which is consistent with our genetic studies in *C. elegans* demonstrating that the ASM homolog is required for the IGF-1R-like signaling pathway under physiological conditions (Kim and Sun, 2012). Importantly, our rescue experiments revealed that externally added ASM on the extracellular leaflet of the plasma membrane is sufficient to regulate the intracellular Golgi transport of the palmitoylated proteins (Fig. 7D). We believe that ASM can produce ceramides on the plasma membrane, which then communicate with the Golgi to regulate the transporting of the palmitoylated proteins. It has been reported that synthetic ceramide analog, C6-ceramide, can be taken up by the cells from culture medium to be transported back to the Golgi (Martin and Pagano, 1994). In our model (shown in Fig. 7F), ASM acts at the outer-leaflet of the plasma membrane to convert sphingomyelins to ceramides. While sphingomyelins are mainly on the outer leaflet of the plasma membrane, ceramides can flip-flop and are therefore present on both leaflets. In addition to the self-association property of ceramides, the removal of the phosphorylcholine head group on the sphingomyelin could also make the otherwise deeply buried cholesterol accessible. Ceramides can retrogradely traffic back to TGN. On the Golgi membrane, ceramides recruit the palmitoylated proteins such as SNAP23 and Lyn to facilitate the transport of these proteins out of the TGN. When ASM is inactivated, SNAP23 and Lyn are trapped in the TGN, due to the lack of the ceramides on the Golgi membrane that are essential for the transport of the palmitoylated proteins out of the TGN. Interestingly, as ASM itself can be found at the plasma membrane in actively growing cells, a location that may allow ASM activity to be regulated by extracellular growth factors, cell attachment and pH environment, which can in turn modulate the function of plasma membrane or the Golgi to govern cell signaling and intracellular Golgi transport processes in a temporally and spatially controlled manner.

## MATERIALS AND METHODS

### Cells, antibodies, and reagents:

Human glioblastoma U373-MG cells were obtained from American Type Culture Collection (ATCC) and were cultured in Dulbecco’s Modified Eagle Medium (DMEM, Invitrogen) supplemented with 10% fetal bovine serum, penicillin and streptomycin at 37°C, 5% CO2. Antibodies for ASM (H-181, sc-11352), Lyn (H-6, sc-7274), Flotillin-2 (clone B-6, sc-28320), VAMP3 (N-12, sc-18208) and Gα(i)2 (L5, sc-13534) were purchased from Santa Cruz Biotechnology; antibodies for SNAP23 (ab4114), Rab14 (ab40938) and GM1 (ab23943) were from Abcam; antibodies for Rab7 (#9367) and Yes (#2734) were from Cell Signaling Technology; antibodies for TGN38 (#610898), GM130 (#610822), CD59 (clone p282, H19), Fyn (clone 25/Fyn) and Fixation and Permeabilization Solution (#554722) were from BD Biosciences; antibodies for ASM/SMPD1 (AF5348, goat IgG), ASM/SMPD1 (MAB5348, mouse IgG2A), and recombinant human ASM/SMPD1 protein (#5348-PD) were from R&D Systems; anti-ceramide mouse monoclonal antibody (clone MID15B4) was from Enzo Life Sciences; anti-Ras (clone 9A11.2, recognizes H, N, K-Ras) mouse monoclonal antibody was from EMD Millipore. Alexa Fluor 488-conjugated goat anti-mouse IgG, 488-conjugated anti-rabbit IgG, Alexa Fluor 647-conjugated anti-mouse IgG, Alexa Fluor 647-conjugated anti-rabbit IgG antibodies, ChromPure Rabbit IgG (011-000-003), ChromPure Mouse IgG (015-000-003), ChromPure Goat IgG (005-000-003) and normal goat serum (005-000-121) were from Jackson ImmunoResearch. Streptavidin-HRP was from Pierce (Thermo Fisher Scientific), protein A and G Sepharose were from GE Healthcare. DAPI (4′, 6-diamidino-2-phenylindole, 32670), desipramine hydrochloride (D3900), 2-bromopalmitate (238422), cerulenin (C2389), cycloheximide and methyl-β-cyclodextrin (MβCD, M7439) were from Sigma-Aldrich.

### Small RNA (siRNA) interference and compound treatment

All siRNAs were synthesized by Dharmacon. Sequences of siRNAs: Human Luciferase siRNA: 5′-CGTACGCGGAATACTTCGA-3′, Human ASM siASM-1: 5′-CTACCTACATC

GGCCTTAA-3′, siRNA-siASM-2: 5′-AGACCTACATCCTGAATCT-3′. U373-MG cells were transfected with 50 nM siRNAs using oligofectamine (Invitrogen) for 48–72 h. For immunostaining, cells were trypsinized at 48 h after transfection and cultured on a 24-well plate for overnight growth. For drug treatment, cells were typically treated with 10 mM Methyl-β-cyclodextrin (MβCD) for 1 h; 25 µM desipramine for 4 h or overnight, 100 µM 2-bromopalmitate for 6 h, and 5 µg/ml cerulenin for 6 h. Immunofluorescence staining and microscopy were conducted as described below. Immunoprecipitation and western blotting were conducted as described previously (Zhang et al., 2013).

### cDNA expression constructs

The cDNA encoding SNAP23 was synthesized and cloned into the pcDNA3.1-N-eGFP vector to derive the pcDNA-N-GFP-SNAP23 construct (service provided by GenScript, Piscataway, NJ, USA). Cys->Ser mutations were subsequently introduced at the amino acid 79, 80, 83, 85 and 87 in the SNAP23 cDNA. pEGFP-N1-human Lyn–GFP was a gift from Anna Huttenlocher (Addgene, plasmid # 35958) (Yoo et al., 2011). Cys->Ser mutations were subsequently introduced at the amino acid 3 and 381 in the Lyn cDNA. Site-directed mutagenesis of SNAP23 and Lyn to introduce Cys->Ser mutations were conducted by GenScript (Piscataway) and all cDNAs were confirmed by sequencing. The expression plasmids were transfected to U373-MG cells by Lipofectamine 2000 and the stable transfectants were obtained following selection for G418 resistance.

### Sphingomyelinase assay

Sphingomyelinase assay was conducted according to described (Horinouchi et al., 1995; Otterbach and Stoffel, 1995). Briefly, cells were lysed in a buffer containing 0.2% Triton X-100, 100 mM sodium acetate, pH 5.2, 1 mM EDTA and with protease inhibitor mixes (Sigma-Aldrich), and sonicated briefly (10 s, three times). Cell debris was pelleted by centrifugation at 13,000 rpm for 5 min at 4°C. Samples were normalized for protein concentration using the Bio-Rad protein assay. The substrate, [Choline-Methyl-^14^C] sphingomyelin (0.02mCi/ml, NEC663010UC, PerkinElmer), was dried and re-suspended in the assay buffer (250 mM sodium acetate, pH 5.2, 1 mM EDTA, 0.2% Triton X-100) and sonicated. Then 100 µl of sphingomyelin substrate and 100 µl of clarified cell lysates were incubated at 37°C for 30–60 min with occasional vortexing. The enzyme reaction was terminated by addition of 0.8 ml of CHCl3: MeOH (2:1, *v/v*) followed by 0.2 ml of water. Samples were vortexed, centrifuged at 5000×***g*** for 95 min, and 0.3 ml of upper aqueous phase was removed for liquid scintillation counting. The assay was linear with time and protein concentration and the maximum substrate hydrolysis was less than 10%.

### Isolation of Brij58-resistant membrane fractions

The Brij-resistant membrane and soluble fractions were isolated according to the described procedure (Giurisato et al., 2003; Montixi et al., 1998; Röper et al., 2000). U373-MG cells grown on the 100 mm tissue culture dishes were washed with ice-cold 1XPBS twice and then incubated with 250 µl ice-cold MBS buffer (25 mM MES, pH6.5, 150 mM NaCl, 50 mM NaF, 2 mM sodium pyrophosphate, 1 mM sodium vanadate and 20 mM β-glycerophosphate) containing 1% Brij58 and the protease inhibitor mixes (10 µg/ml aprotinin, 1 mM benzamidine and 10 µg/ml leupeptin). Cells were then scraped off the dish and homogenized with a Dounce homogenizer (1 ml) with 20 strokes. Two 100 mm dishes of cells were used for each sample. Cell debris was clarified by centrifugation at 960×***g*** for 10 min at 4°C and the clarified cell extracts were normalized for equal amount of total proteins in each sample in 500 µl total volume. The lysates were mixed with equal volume (500 µl) of 80% sucrose in the MBS buffer (sucrose-MBS) with 1% Brij58 to obtain 40% final sucrose concentration. This mixture was loaded under the 3.2 ml of 35% sucrose-MBS in the centrifuge tube, and an additional 0.80 ml of 5% sucrose-MBS was added slowly on top of 35% sucrose-MBS to form a discontinuous sucrose gradient of 5%, 35% and 40% for buoyant density gradient ultracentrifugation. The sucrose gradients were centrifuged in a Beckman SW55 Ti swinging rotor at 41,000 rpm, 4°C, for 18 h. After centrifugation, 300 µl per fraction was taken from the top (low density, fraction #1) to the bottom (high density, fraction #16) of the centrifuge tube. The Brij58-resistant membrane fractions were isolated as a band from the 5% to 35% sucrose interference (fractions #1–4), where Brij58-soluble fractions were in the bottom fractions (fractions #13–16). By Bradford protein concentration determination, total protein concentration in pooled fractions of #1–4 was only 1% of the total protein concentration in the pooled fractions of #13–16. Proteins associated with the fractions were analyzed by western blotting with specific antibodies or processed for mass spectrometry proteomic analysis.

### Protein gel electrophoresis and in-gel trypsin digestion

Fraction #1–4 from the ultracentrifugation were pooled, and concentrated by Amicon Ultra centrifugal filters (10 kDa cut-off, Millipore) then resolved on a 4–12.5% gradient Nu-PAGE gel (Invitrogen). Proteins were stained with Coomassie Blue, and extracted from the gel by first slicing the gel into 24 equally spaced gel pieces from the bottom to the top of the gel (10–300 kDa). Individual gel slices were de-stained with 25 mM NH_4_HCO_3_ in 50% acetonitrile (ACN), reduced with 20 mM dithiothreitol in 25 mM NH_4_HCO_3_ for 1 h at 37°C and alkylated with 55 mM iodoacetamide in 25 mM NH_4_HCO_3_ in the dark for 1 h at room temperature. After washing, the gel slices were dehydrated with ACN and dried using a Speed-Vacuum system. Mass spectrometry-grade trypsin (Promega) was incubated with each gel slice overnight at 37°C. The supernatant was collected and dried using a Speed-Vacuum for subsequent liquid chromatography mass spectrometry (LC-MS/MS) analysis.

### Mass spectrometry-based proteomic analysis of proteins

Tryptic peptides derived from each gel slice were analyzed by an on-line C18 nano-flow reversed-phase liquid chromatography instrument (Easy nano-liquid chromatography) connected to an LTQ Orbitrap XL mass spectrometer (Thermo Fisher Scientific). Samples were concentrated onto an in-house packed 100-nm-inner diameter×1-cm C18 column (Magic C18, 5 µm, 300Å, Michrom Bioresources Inc.) then separated on 50-nm-inner diameter×15-cm C18 column at 300 nl/min with 75 min linear gradients from 0 to 35% acetonitrile in 0.1% formic acid. The LC eluent was directly nanosprayed into an LTQ Orbitrap XL mass spectrometer (Thermo Fisher Scientific) with an ionization voltage of 2.2 KV. During the chromatographic separation, the LTQ Orbitrap XL is operated in a data-dependent mode and under the direct control of Xcalibur (Thermo Fisher Scientific). The mass spectrometry (MS) data were acquired using the following parameters: 5 data-dependent collision-induced dissociation MS2 scan survey in the linear ion trap per every full scan in the Orbitrap with the resolution set to a value of 60,000; 35% normalized collision energy in CID; ±2 Da isolation window.

### Proteomic data analysis

Proteomic profiling data were first analyzed by QualBrowser in Xcalibur and Proteome Discoverer to identify proteins with unique peptides, and followed by analysis with the Scaffold software (Proteome Software, Portland, OR, USA), which uses advanced algorithms to improve statistical significance. Only proteins that passed the protein probability threshold above 99%, peptide probability threshold above 95% and contained at least one unique peptide were taken for label-free quantitation. Total number of high quality peptides identified in the DMSO control or desipramine-treated samples are 3345 and 3644, respectively, which were then subjected to spectra counting. The spectrum number from each experiment was exported to Excel spreadsheets and the relative abundance of proteins were calculated based on the published protocols. Details are described below.

The Thermo .raw files were analyzed by Proteome Discoverer (version 1.0) which searched against IPI human database (v3.75; 89,486 sequences) using following parameters: two tryptic mass cleavages, 10 ppm precursor ion mass tolerance, 0.8 Da fragment ion mass tolerance, fix modification of carbamidomethyl on cysteines, and variable protein modification of oxidation on methionines. For protein identification and label-free quantitation, the .msf files generated from Proteome Discoverer were subsequently analyzed by Scaffold 3 software (version 3.0; Proteome Software Inc.).

Results were filtered on Scaffold to achieve a protein probability <1.0% and peptide probability <5.0% with at least one unique peptide identification. The spectra counts were used to calculate the relative abundance of proteins were calculated based on the published protocols (Liu et al., 2004; Old et al., 2005): R_SC_=log2 [(n2+f)/(n1+f)]+log2[(t1-n1+f)/(t2-n2+f)], where, for each protein, R_SC_ is the log2 ratio of abundance between samples 1 and 2; n1 and n2 are spectral counts for the protein in samples 1 and 2, respectively; t1 and t2 are total numbers of spectra over all proteins in the two samples; and ƒ is a correction factor set to 1.25. The candidate ASM-dependent membrane-associated proteins are defined as the proteins that were sensitive to ASM siRNA using a threshold of 2.0 and at least two unique peptides, and this group of proteins is defined as the high confidence group. In addition, there are a group of proteins which contains at least one unique peptide, with a change of protein abundance threshold of 1.8, and this group of proteins is considered low confidence group.

### Immunofluorescence microscope image acquisition

Cells were cultured on 24-well plate for 24 h and then the cells were transfected with specific ASM siRNAs or treated with specific chemical compound for indicated times. Cells were washed briefly with PBS and immediately fixed with BD Fixation and Permeabilization Solution for 30 min. After incubated with the primary antibodies overnight at 4°C, cells were washed in PBS and incubated with fluorescence labeled secondary antibodies or with biotin-streptavidin system at room temperature. Cells were staining with PBS containing 1 μg/ml DAPI for DNA counter staining. Images were captured by an automated confocal microscope system with high content screen capability, Opera™ LX (Perkin Elmer Inc.) with 24× (NA1.0) or 40× (NA1.1) water immersion lenses. Images were processed by using the on-board Acapella software. Alternatively, images were acquired by Nikon A1R+ confocal microscope using 40× oil immersion lens (NA1.3), as previously described (Zhu et al., 2016). Quantitations of the relative proteins abundance on the plasma membrane are carried out using the fluorescence images and ImageJ software as described (Zhu et al., 2016). ChromPure Rabbit IgG or ChromPure Mouse IgG were used as negative controls.

### The ABE assay for palmitoylated proteins

1×10^8^ active growing U373-MG cells were collected by centrifugation and then re-suspended in 3 ml ice-cold lysis buffer containing 10 mM N-Ethylmaleimide (NEM, freshly diluted from 1M stock prepared in ethanol), 2× protease inhibitor mix (Sigma-Aldrich) and 2 mM phenylmethylsulfonyl fluoride (PMSF), and then homogenized with 25 strokes of a Dounce homogenizer. Triton X-100 was added to 1.7% and the lysate was incubated with gentle mixing for 1 h at 4°C. Cell debris was removed by centrifugation at 5000 rpm for 10 min. The ABE assays were performed according to (Kang et al., 2008; Roth et al., 2006). Briefly, proteins were precipitated by a chloroform-methanol mixture and then treated extensively with N-Ethylmaleimide (NEM) to block free thiol groups. After reaction, free NEM was removed by sequential chloroform-methanol (CM) precipitation. In the NEM-blocked lysates, the palmitoyl group of palmitoylated proteins is removed from the palmitoylated thio- groups of cysteine residues by treating the lysates with or without hydroxylamine (HA). The HA-exposed thio- groups were labeled with biotin-HPDP and the free biotin-HPDP is removed by precipitation. The precipitated proteins were solubilized and the biotin-HPDP modified proteins were incubated with streptavidin-agarose resins. The bound proteins on the streptavidin-agarose beads were extensively washed (>four times) with the lysis buffer and then bound proteins were separated in SDS-PAGE. Specific proteins in the streptavidin-agarose beads were identified by western blotting with specific antibodies.

### Statistical analysis

Experiments were usually performed with at least three independent repeats (biological replicates) to ensure the results. For cell number assays, triplicated repeats in the same set of cells were measured and the experiments usually repeated in three independent experiments with different cultured cells. Quantitative data are expressed by bar graph and standard deviations (s.d.) are expressed as mean and error bars. For siRNA-mediated knockdown or drug treatment experiments, statistically significant differences between means of control and knockdown/drug treated were compared using a two-tailed equal-variance independent *t*-test (Fay and Gerow, 2013). Different data sets were considered to be statistically significant when the *P*-value was <0.01.

## Supplementary Material

Supplementary information
