# Supplementary material for: Acid sphingomyelinase regulates the localization and trafficking of palmitoylated proteins
Source: Biol Open. 2019 May 29;8(10):bio040311. doi: 10.1242/bio.040311 (PMC6826292; doi:10.1242/bio.040311)
Supplement: Supplementary information [file biolopen-8-040311-s1.pdf]

Table S1. The spectral counting data for the protein hits that are decreased in the DRM association following ASM siRNA treatment.

U373-MG cells were treated with the luciferase siRNAs (siLuc) as control or with ASM specific siRNAs (siASM). DRM fractions from both samples were processed and analyzed by OrbiTrap mass-spectrometer. Proteomic data were analyzed by Proteome Discoverer software and Scaffold software. The filtered spectra counts were quantitated according to (Liu et al., 2004; Old et al., 2005) to derive the R_{SC}, which is the log2 ratio of abundance between Samples 1 and 2. The R_{SC} score was then used to calculate the ratio of abundance between Samples 1 (siLuc) and 2 (siASM). Fold of protein abundance change between control siLuc and siASM, as well as numbers of the peptides identified in each condition, are listed in the middle columns. The high confidence group are consisted of proteins each identified by at least two unique peptides, and the ratio of abudundance change >2. The low confidence group are consisted of proteins each identified by at least one unique peptides, and the ratio of abudance change >1.8.

Bio View:Identified Proteins (878)	Accession Number	siLuc # peptides	siASM # peptides	siLuc/siASM Fold of Change
High Confidence, number of unique peptides number >2, fold of change >2.0, from siLuc/siASM comparison				
CD59 glycoprotein, T cell activation	IPI00011302	44	0	36.2
Putative uncharacterized protein ZNF326	IPI00337602 (+1)	71	1	32.1
VAMP3, Vesicle-associated membrane protein 3	IPI00549343	37	0	30.6
Vesicle-trafficking protein SEC22b	IPI00006865	28	0	23.4
Beta-2-microglobulin	IPI00004656 (+1)	19	0	16.2
Caveolin, scaffolding protein within caveolar membranes	IPI00009236	63	4	12.2
5'-nucleotidase ecto, CD73	IPI00009456	14	0	12.2
ATP synthase subunit alpha, mitochondrial	IPI00440493	13	0	11.4
RPN2, subunit of N-oligosaccharyl transferase complex	IPI00028635 (+1)	12	0	10.6
VAMP7, vesicle-associated membrane protein 7, Isoform 1	IPI00020887	10	0	9
60 kDa heat shock protein, mitochondrial	IPI00784154	8	0	7.4
Ras-related protein Rab-7a	IPI00016342	7	0	6.6
Src family tyrosine kinase Yes	IPI00013981	7	0	6.6
Syntaxin-16, Isoform B	IPI00023149 (+1)	7	0	6.6
RPN1, subunit of N-oligosaccharyl transferase complex	IPI00025874	7	0	6.6
Glypican-4	IPI00232571	20	2	6.5
CD90, possible cell-cell or cell-ligand interaction,Thy-1 membrane glycoprotein	IPI00022892 (+1)	18	2	5.9
Major prion protein, Isoform 1	IPI00022284 (+4)	12	1	5.9
Ras-related protein Rab-1A, isoform 1	IPI00005719 (+1)	6	0	5.8
GCP16, palmitoyltransferase, protein palmitoylation	IPI00480022 (+1)	6	0	5.8
Putative receptor for complement component 1 Q, mitochondrial	IPI00014230	6	0	5.8
Annexin A2, Isoform 2	IPI00418169 (+1)	16	2	5.3
T-complex protein 1 subunit beta	IPI00297779	5	0	5
Ragulator complex protein LAMTOR1, mTOR regulation	IPI00016670	5	0	5
Ras-related protein R-Ras	IPI00020418	5	0	5
Myeloid-associated differentiation marker	IPI00102685 (+1)	5	0	5
Serine/threonine-protein kinase Nek2, Isoform 1	IPI00021331	20	3	5
Cytochrome c oxidase subunit 5A, mitochondria	IPI00025086	5	0	5
Ras-related protein Ral-B	IPI00004397	5	0	5
Erythrocyte band 7 integral membrane protein	IPI00219682	18	3	4.5
Ras-related protein Rab-5C	IPI00016339	4	0	4.2
Proteolipid protein 2, function unknown	IPI00030362	4	0	4.2
Protein FAM3C	IPI00334282	4	0	4.2
Sodium-dependent amino acids transporter	IPI00019472	4	0	4.2
Limbic system-associated membrane protein	IPI00013303	4	0	4.2
SNAP23, Synaptosomal-associated protein 23	IPI00010438	4	0	4.2
Pyruvate kinase PKM, glycolysis	IPI00479186	4	0	4.2

GTPase IMAP family member 8	IPI00168482	4	0	4.2
Cytochrome b-c1 complex subunit Rieske, mitochondrial	IPI00026964	4	0	4.2
ATP synthase subunit delta, mitochondrial	IPI00024920	4	0	4.2
91 kDa protein	IPI00871370 (+1)	4	0	4.2
Heat shock cognate 71 kDa protein,iIsoform 1	IPI00003865	15	3	3.8
Peroxidasin homolog, isoform 1	IPI00016112	26	6	3.8
Secretory carrier-associated membrane protein 1, Isoform 1	IPI00005129	10	2	3.5
Transmembrane emp24 domain-containing protein 10	IPI00028055	3	0	3.4
T-complex protein 1 subunit eta	IPI00018465 (+3)	3	0	3.4
Synaptogyrin-2	IPI00013946 (+2)	3	0	3.4
Stress-70 protein, mitochondrial	IPI00007765	3	0	3.4
Ras-related protein Rap-2b	IPI00018364	3	0	3.4
Ras-related protein Ral-A	IPI00217519	3	0	3.4
Protein FAM162A	IPI00023001 (+1)	3	0	3.4
Protein disulfide-isomerase PDI, protein folding	IPI00010796	3	0	3.4
Mitogen-activated protein kinase scaffold protein 1	IPI00030919	3	0	3.4
Reticulon-3, isoform 2	IPI00398795 (+3)	3	0	3.4
CD97 antigen, Isoform 2, potential cell adhesion receptor	IPI00299412 (+4)	3	0	3.4
IFM3, Interferon-induced transmembrane protein 3	IPI00303726	3	0	3.4
Deoxyribonuclease-1-like 1	IPI00026125 (+1)	3	0	3.4
Cytochrome c oxidase subunit 6B1	IPI00216085 (+1)	3	0	3.4
Cytochrome c oxidase subunit 4 isoform 1, mitochondrial	IPI00006579	3	0	3.4
Cofilin-1, actin cytoskeleton regulation	IPI00012011	3	0	3.4
Choline-phosphate cytidylyltransferase A	IPI00329338 (+1)	3	0	3.4
cDNA FLJ59191, highly similar to NADH dehydrogenase (ubiquinone) 1 alpha subcomplex subunit 13	IPI00942935	3	0	3.4
T-complex protein 1 subunit theta	IPI00302925 (+1)	3	0	3.4
Syntaxin-10, isoform 1	IPI00293402 (+1)	6	1	3.2
Prohibitin	IPI00017334	8	2	2.8
Transporter for monocarboxylate, metabolism	IPI00024650	8	2	2.8
Cell sruface receptor, cell-matrix interaction	IPI00297160 (+19)	22	7	2.8
Ga(i)2, isoform 1, heterotrimeric G protein subunit, isoform 1	IPI00748145	5	1	2.8
Collapsin response mediator protein 4 long variant	IPI00029111	23	8	2.6
T-complex protein 1 subunit delta	IPI00302927 (+2)	2	0	2.6
Stomatin-like protein 2	IPI00334190	2	0	2.6
Ras-related protein Rab-2A	IPI00031169	2	0	2.6
Ras-related protein Rab-14	IPI00291928	2	0	2.6
Subunit of succinate dehydrogenase complex, mitochondrial	IPI00965327	2	0	2.6
Putative chloride channel protein 7	IPI00020524 (+2)	2	0	2.6
Protein disulfide-isomerase A3	IPI00025252	2	0	2.6
Profilin-1	IPI00216691	2	0	2.6
Peptidyl-prolyl cis-trans isomerase A, protein folding	IPI00419585	2	0	2.6
monocarboxylate transporter 8	IPI00000655	2	0	2.6
MHC class I antigen	IPI00794678 (+2)	2	0	2.6
Lysosomal acid phosphatase	IPI00003807	2	0	2.6

Biology Open • Supplementary information

Src familty tyrosine kinase Fyn, isoform 3	IPI00166845 (+2)	2	0	2.6
Vesicle-associated membrane protein-associated protein B/C, isoform 1	IPI00006211	2	0	2.6
Uncharacterized protein C4orf52, isoform 1	IPI00397994 (+1)	2	0	2.6
Solute carrier family 12 member 2, isoform 1	IPI00022649 (+1)	2	0	2.6
Ras-related protein Rab-6A, Isoform 1	IPI00023526 (+1)	2	0	2.6
Ovarian carcinoma immunoreactive antigen-like protein	IPI00555902	2	0	2.6
Mitogen-activated protein-binding protein-interacting protein,iIsoform 1	IPI00032409	2	0	2.6
GDNF family receptor alpha-1, isoform 1	IPI00008148 (+1)	2	0	2.6
Elongation factor 1-delta, isoform 1	IPI00023048 (+2)	2	0	2.6
Electron transfer flavoprotein subunit beta, isoform 1	IPI00004902 (+1)	2	0	2.6
E3 ubiquitin-protein ligase CHIP, isoform 1	IPI00025156 (+1)	2	0	2.6
Apoptosis-inducing factor 1, Isoform 1, mitochondrial	IPI00000690 (+1)	2	0	2.6
Integral membrane protein 2B, amyloid precursor processing	IPI00031821	2	0	2.6
Hippocalcin-like protein 1, calcium binding and sensing	IPI00219344	2	0	2.6
Ga(i)3, G(k) subunit alpha, heterotrimeric G protein subunit	IPI00220578	2	0	2.6
Guanine nucleotide-binding protein G(I)/G(S)/G(O) subunit gamma-12	IPI00221232	2	0	2.6
MICOS complex subunit MIC19, mitochondrial	IPI00015833 (+4)	2	0	2.6
CD81, Tetraspanin-28, cell surface protein	IPI00000190 (+1)	2	0	2.6
Calmodulin	IPI00075248 (+3)	2	0	2.6
Calcium-binding mitochondrial carrier protein Aralar2	IPI00007084 (+1)	2	0	2.6
B-cell receptor-associated protein 31	IPI00218200 (+1)	2	0	2.6
ATP synthase subunit b, mitochondrial	IPI00029133	2	0	2.6
Src family tyrosine kinase Lyn, isoform B	IPI:IPI00432416.4	2	0	2.6
Annexin A1	IPI00218918	2	0	2.6
Sortilin	IPI00217882	7	2	2.5
Ephrin type-A receptor 2, receptor tyrosine kinase	IPI00021267	4	1	2.3
Secretory carrier-associated membrane protein 3, isoform 1	IPI00306382	18	8	2.1
Low Confidence, Number of unique peptides > 1, fold of change > 1.8, from siLuc/siASM comparison				
Syntaxin-7, isoform 1	IPI00289876 (+1)	11	5	1.96
Transmembrane protein C2orf18	IPI00550440 (+2)	5	2	1.9
Dolichyl-diphosphooligosaccharide--protein glycosyltransferase 48 kDa subunit	IPI00297084	5	2	1.9
V-type proton ATPase subunit d 1	IPI00034159	3	1	1.9
Translation initiation factor eIF-2B subunit epsilon	IPI00011898 (+1)	3	1	1.9
ATP synthase subunit gamma, Isoform liver, mitochondrial	IPI00478410	3	1	1.9
Seprase, isoform 1	IPI00295461 (+1)	3	1	1.9
Hormonally up-regulated neu tumor-associated kinase	IPI00219553 (+1)	3	1	1.9
14-3-3 protein gamma	IPI00220642	3	1	1.9
Vesicle transport through interaction with t-SNAREs homolog 1A	IPI00059472 (+1)	1	0	1.8
Vacuolar protein sorting-associated protein 45	IPI00090327	1	0	1.8
V-type proton ATPase subunit B, brain isoform	IPI00007812	1	0	1.8
V-type proton ATPase 116 kDa subunit a isoform 2	IPI00000425	1	0	1.8
tumor protein D54 isoform a	IPI00399265 (+4)	1	0	1.8
tropomyosin alpha-3 chain isoform 1	IPI00183968 (+7)	1	0	1.8

Biology Open • Supplementary information

Translocator protein	IPI00026850	1	0	1.8
Transgelin-2	IPI00550363 (+1)	1	0	1.8
TMEM181 protein (Fragment)	IPI00166790 (+1)	1	0	1.8
T-complex protein 1 subunit zeta	IPI00027626	1	0	1.8
T-complex protein 1 subunit gamma isoform b	IPI00290770 (+3)	1	0	1.8
Sodium/potassium-transporting ATPase subunit beta-3	IPI00008167	1	0	1.8
Sideroflexin-1	IPI00009368	1	0	1.8
Regulator of G-protein signaling 19	IPI00028108	1	0	1.8
Ras-related protein Rap-1b	IPI00015148	1	0	1.8
Ras-related protein Rab-11B	IPI00020436	1	0	1.8
Prolactin-inducible protein	IPI00022974	1	0	1.8
Podocalyxin-like protein 1 precursor	IPI00299116 (+2)	1	0	1.8
Peptidyl-prolyl cis-trans isomerase B	IPI00646304	1	0	1.8
NADH dehydrogenase [ubiquinone] iron-sulfur protein 8, mitochondrial	IPI00010845	1	0	1.8
NADH dehydrogenase [ubiquinone] flavoprotein 2, mitochondrial	IPI00291328 (+2)	1	0	1.8
NADH dehydrogenase [ubiquinone] 1 beta subcomplex subunit 10	IPI00479905	1	0	1.8
NADH dehydrogenase [ubiquinone] 1 alpha subcomplex subunit 5	IPI00554681	1	0	1.8
Mitochondrial import inner membrane translocase subunit Tim23	IPI00007309 (+5)	1	0	1.8
Matrix metalloproteinase-14	IPI00218398 (+1)	1	0	1.8
Vesicle transport through interaction with t-SNAREs homolog 1B, isoform Long	IPI00063784	1	0	1.8
Protein SON, isoform F	IPI00000192 (+3)	1	0	1.8
Integrin alpha-6, isoform Alpha-6X1X2B	IPI00010697 (+6)	1	0	1.8
Zinc finger protein 781, isoform 1	IPI00853563	1	0	1.8
Voltage-dependent anion-selective channel protein 3, isoform 1	IPI00031804 (+1)	1	0	1.8
Transmembrane protein 87B, isoform 1	IPI00783380 (+1)	1	0	1.8
Transmembrane protein 55B, isoform 1	IPI00030530 (+1)	1	0	1.8
Trans-2,3-enoyl-CoA reductase, isoform 1	IPI00100656	1	0	1.8
TBC domain-containing protein kinase-like protein, isoform 1	IPI00291665	1	0	1.8
Secretory carrier-associated membrane protein 4, isoform 1	IPI00056310 (+2)	1	0	1.8
Pyruvate dehydrogenase E1 component subunit beta, isoform 1, mitochondrial	IPI00003925 (+2)	1	0	1.8
Putative adenosylhomocysteinase 2, isoform 1	IPI00182938 (+2)	1	0	1.8
Phosphatidylinositol 4-kinase alpha, isoform 1	IPI00070943	1	0	1.8
Mitochondrial Rho GTPase 2, isoform 1	IPI00465059	1	0	1.8
Exportin-2, isoform 1	IPI00022744 (+1)	1	0	1.8
Erlin-2, isoform 1	IPI00026942	1	0	1.8
Cytoskeleton-associated protein 4, isoform 1	IPI00141318 (+1)	1	0	1.8
Cell cycle control protein 50A, isoform 1	IPI00019381 (+2)	1	0	1.8
ATP synthase subunit d, isoform 1, mitochondrial	IPI00220487	1	0	1.8
FAM73A protein	IPI00168047	1	0	1.8
Dystroglycan	IPI00028911	1	0	1.8
Dihydrolipoyl dehydrogenase, mitochondrial	IPI00015911	1	0	1.8
cytochrome c oxidase subunit VIIa polypeptide 2 (liver) precursor	IPI00026570 (+1)	1	0	1.8
Cytochrome c oxidase subunit 6C	IPI00015972	1	0	1.8
Cytochrome c oxidase subunit 5B, mitochondrial	IPI00021785	1	0	1.8

Biology Open • Supplementary information

Cytochrome c oxidase subunit 2	IPI00017510	1	0	1.8
cDNA FLJ56280, highly similar to Endoplasmic reticulum-Golgi intermediate compartment protein 1	IPI00003635 (+1)	1	0	1.8
cDNA FLJ56157, highly similar to Glucosylceramidase	IPI00021807 (+2)	1	0	1.8
Calcyphosin	IPI00792011 (+1)	1	0	1.8
ATP synthase subunit O, mitochondrial	IPI00007611	1	0	1.8
ADP/ATP translocase 1	IPI00022891	1	0	1.8
7-dehydrocholesterol reductase	IPI00294501	1	0	1.8

Table S2. The spectral counting data for the protein hits that are increased in the DRM association following ASM siRNA treatment.

Experiments, data analyses and quantitation were conducted similar as in the legends of Supplementary Table 1. Shown are the proteins which show abundance change greater than >2-fold between the sample 2 (siASM) and Samples 1 (siLuc). Fold of protein abundance change between siASM and control siLuc, as well as numbers of the peptides identified in each condition, are listed in the middle columns.

Bio View:Identified Proteins (878)	Accession Number	siLuc # peptides	siASM # peptides	siASM/siLuc Fold of Change
High Confidence, number of unique peptides number >2, fold of change >2.0, from siASM/siLuc comparison				
Myoferlin, isoform 1	IPI00021048 (+1)	1	101	45.4
Flotillin-1	IPI00027438	1	86	38.8
Flotillin-2	IPI00789008	1	61	27.7
Alpha-enolase, isoform alpha	IPI00465248	0	27	22.6
Moesin	IPI00219365 (+1)	0	24	20.2
Reticulon-4, isoform 2	IPI00298289	0	21	17.8
Golgi apparatus protein 1,isoform 2	IPI00414717 (+2)	0	19	16.2
Mitochondrial inner membrane protein, isoform 2	IPI00554469	0	17	14.6
Inositol 1,4,5-trisphosphate receptor type 3	IPI00291607	0	14	12.2
Vesicle-fusing ATPase	IPI00006451 (+1)	0	12	10.6
Cytochrome b-c1 complex subunit 1, mitochondrial	IPI00013847	2	31	9.9
NADH dehydrogenase [ubiquinone] iron-sulfur protein 2, mitochondrial	IPI00025239 (+1)	0	11	9.8
Apolipoprotein B-100	IPI00022229	0	11	9.8
Trifunctional enzyme subunit beta, mitochondrial	IPI00022793	0	10	9
Cathepsin D	IPI00011229	0	10	9
Heat shock protein HSP 90-beta	IPI00414676	0	9	8.2
Cytochrome b-c1 complex subunit 2, mitochondrial	IPI00305383	2	25	8.1
Metalloreductase STEAP3, isoform 1	IPI00019350 (+3)	0	8	7.4
Cation-dependent mannose-6-phosphate receptor	IPI00025049	0	7	6.6
Phosphoglycerate kinase 1	IPI00169383	2	18	5.9
V-type proton ATPase 116 kDa subunit a isoform 1, isoform 2	IPI00743576 (+2)	2	18	5.9
DNA-dependent protein kinase catalytic subunit, isoform 1	IPI00296337	0	6	5.8
Carboxypeptidase D	IPI00027078	3	22	5.5
Vimentin	IPI00418471	0	5	5
Very low-density lipoprotein receptor, isoform Long	IPI00024273 (+2)	2	15	5
Integrin beta-4, Isoform Beta-4C	IPI00027422 (+3)	0	5	5
Integrin beta-3, Isoform Beta-3A	IPI00303283	0	5	5
Calcium-transporting ATPase type 2C member 1, isoform 2	IPI00220473 (+8)	0	5	5
Surfeit locus protein 4, isoform 1	IPI00005737 (+2)	0	5	5
Plexin-D1, isoform 1	IPI00412492 (+1)	0	5	5
Epoxide hydrolase 1	IPI00009896	0	5	5
Collagen alpha-3(V) chain	IPI00018279	0	5	5
Conserved hypothetical protein	IPI00916096	7	38	4.8
Neuroblast differentiation-associated protein AHNAK	IPI00021812	1	9	4.6
LAMP-2A of Lysosome-associated membrane glycoprotein 2	IPI00009030 (+2)	1	9	4.6
Endoplasmin	IPI00027230	1	9	4.6
Sulfide:quinone oxidoreductase, mitochondrial	IPI00009634	0	4	4.2

Biology Open • Supplementary information

Protein disulfide-isomerase A4	IPI00009904	0	4	4.2
Desmoglein-4, isoform 2	IPI00428691 (+1)	0	4	4.2
Tenascin, isoform 1	IPI00031008 (+6)	0	4	4.2
Leucyl-cystinyl aminopeptidase, isoform 1	IPI00307017 (+1)	0	4	4.2
Heterogeneous nuclear ribonucleoprotein M, isoform 1	IPI00171903 (+1)	0	4	4.2
Gelsolin, isoform 1	IPI00026314 (+2)	0	4	4.2
High affinity cationic amino acid transporter 1	IPI00027728	0	4	4.2
Ceruloplasmin	IPI00017601 (+1)	0	4	4.2
Calpain-1 catalytic subunit	IPI00011285	0	4	4.2
A-kinase anchor protein 6	IPI00297089	0	4	4.2
Niemann-Pick C1 protein	IPI00005107	1	8	4.1
Translational activator GCN1	IPI00001159	1	7	3.7
signal-regulatory protein alpha precursor	IPI00332887 (+1)	0	3	3.4
Serotransferrin	IPI00022463	0	3	3.4
Prostaglandin F2 receptor negative regulator	IPI00022048	0	3	3.4
Prolow-density lipoprotein receptor-related protein 1	IPI00020557	0	3	3.4
NudC domain-containing protein 2	IPI00103142	0	3	3.4
TGN51 of Trans-Golgi network integral membrane protein 2	IPI00012545 (+4)	0	3	3.4
Gnas-2 of Guanine nucleotide-binding protein G(s) subuni, alpha isoforms short	IPI00219835 (+1)	0	3	3.4
Perilipin-3, isoform B	IPI00303882	0	3	3.4
Adenylate kinase domain-containing protein 1, isoform 6	IPI00552962 (+1)	0	3	3.4
Protein disulfide-isomerase A6, isoform 2	IPI00299571 (+1)	0	3	3.4
Sodium/potassium-transporting ATPase subunit beta-1, isoform 1	IPI00747849 (+1)	0	3	3.4
Heparan sulfate 2-O-sulfotransferase 1, isoform 1	IPI00549891	0	3	3.4
Choline transporter-like protein 2, isoform 1	IPI00549521	0	3	3.4
CD276 antigen, isoform 1	IPI00410488 (+3)	0	3	3.4
Catenin beta-1, isoform 1	IPI00017292 (+1)	0	3	3.4
Calcium-binding mitochondrial carrier protein SCaMC-1, isoform 1	IPI00337494	0	3	3.4
Integrin alpha-2	IPI00013744	0	3	3.4
HCG1990625, isoform CRA_a	IPI00969623	0	3	3.4
cDNA FLJ56903, highly similar to Tubulin beta-7 chain	IPI00909140	0	3	3.4
cDNA FLJ56389, highly similar to Elongation factor 1-gamma	IPI00000875 (+1)	0	3	3.4
cDNA FLJ55574, highly similar to Calnexin	IPI00020984 (+1)	8	30	3.4
Transferrin receptor protein 1	IPI00022462	21	73	3.3
NADH-ubiquinone oxidoreductase 75 kDa subunit	IPI00604664 (+1)	1	6	3.2
C-type mannose receptor 2	IPI00005707	1	6	3.2
Transmembrane protein 87A, isoform 1	IPI00783698 (+1)	3	11	2.9
Voltage-dependent anion-selective channel protein 2, isoform 2	IPI00024145 (+4)	2	8	2.8
DnaJ homolog subfamily C member 13	IPI00307259	1	5	2.8
Protein S100-A9	IPI00027462 (+1)	0	2	2.6
proteasome-associated protein ECM29 homolog	IPI00157790	0	2	2.6
N-acetylglucosamine-6-sulfatase	IPI00012102 (+1)	0	2	2.6
Leucine-rich PPR motif-containing protein, mitochondrial	IPI00783271	0	2	2.6
Neurofascin, Isoform 7	IPI00384998 (+11)	0	2	2.6

Biology Open • Supplementary information

nhibitor of nuclear factor kappa-B kinase-interacting protein, isoform 4	IPI00043598	0	2	2.6
Inositol 1,4,5-trisphosphate receptor type 1, isoform 3	IPI00218659 (+4)	0	2	2.6
Semaphorin-4B, isoform 2	IPI00513964	0	2	2.6
Multidrug resistance-associated protein 1, isoform 2	IPI00008338 (+6)	0	2	2.6
Dynein heavy chain 2, axonemal, isoform 2	IPI00651691 (+1)	5	15	2.6
Titin, isoform	IPI00759754 (+4)	0	2	2.6
Putative SMEK homolog 3, isoform 1	IPI00900377	0	2	2.6
Nicastrin, isoform 1	IPI00021983 (+1)	0	2	2.6
NADH dehydrogenase [ubiquinone] flavoprotein 1, mitochondrial, isoform 1	IPI00028520 (+1)	0	2	2.6
Kinectin, isoform 1	IPI00328753	0	2	2.6
Glial fibrillary acidic protein, isoform 1	IPI00025363 (+2)	0	2	2.6
Epidermal growth factor receptor, isoform 1	IPI00018274	0	2	2.6
Inositol monophosphatase 3	IPI00787853	0	2	2.6
HLA class I histocompatibility antigen, alpha chain G	IPI00015988 (+3)	0	2	2.6
Histone H3.2	IPI00171611 (+6)	0	2	2.6
Fatty acid-binding protein, epidermal	IPI00007797	0	2	2.6
Ezrin	IPI00843975 (+1)	0	2	2.6
Dynein heavy chain domain-containing protein 1	IPI00936051	0	2	2.6
cDNA FLJ60299, highly similar to Rab GDP dissociation inhibitor beta	IPI00031461 (+1)	0	2	2.6
Alpha-mannosidase 2	IPI00003802	0	2	2.6
60S ribosomal protein L4	IPI00003918	0	2	2.6
Transmembrane 9 superfamily member 4	IPI00021985 (+1)	2	7	2.5
NADPH--cytochrome P450 reductase	IPI00470467 (+3)	1	4	2.3
NADH-cytochrome b5 reductase 1	IPI00470674	1	4	2.3
Neuroplastin, isoform 1	IPI00011578 (+4)	1	4	2.3
CD166 antigen, isoform 1	IPI00015102 (+3)	1	4	2.3
Acylglycerol kinase, isoform 1, mitochondrial	IPI00019353 (+1)	1	4	2.3
cDNA FLJ52398, highly similar to Cadherin-13	IPI00024046	1	4	2.3
Voltage-dependent anion-selective channel protein 1	IPI00216308	8	20	2.3
Trifunctional enzyme subunit alpha, mitochondrial	IPI00031522	2	6	2.2
Monocarboxylate transporter 4	IPI00006666	2	6	2.2
Inner centromere protein, isoform 2	IPI00759472 (+1)	2	6	2.2
51 kDa protein	IPI00328883 (+8)	2	6	2.2
HLA class I histocompatibility antigen, A-2 alpha chain	IPI00876963 (+3)	10	23	2.2
Lysosome membrane protein 2	IPI00217766	6	14	2.1

Biology Open • Supplementary information

Table S3. List of proteins identified in ASM-sensitive DRM proteome that are also known to be palmitoylated proteins.

Hits listed in supplementary Table 1 were cross-referenced to the palmitoyl-proteome database (SwissPalm) (Blanc et al., 2015), 65 proteins are found to be known as palmitoylated protein and listed here. Semi-quantitative mass-spectrometry data, including the fold of protein abundance change between cells treated with Luciferase siRNA (siLuc) and ASM siRNA (siASM), as well as the numbers of the peptides identified in each condition for each protein, are shown in the middle columns. The numbers of articles that reported the particular protein found in the palmitoyl-proteomes is indicated in the second to the last column. Both UniProt accession number and IPI accession numbers are listed. Proteins are grouped according to their functional categories.

UniProt accession number	Protein name	Protein Functions or Biological Process	Fold of change	siLuc peptides	siASM peptides	# of articles palmitoy-proteome studies	IPI database Accession Number
High Confidence, number of unique peptides number >2, fold of change >2.0							
Receptors, Cell surface proteins or transporters:							
P13987	CD59	CD59 glycoprotein, T cell activation	36.2	44	0	3 of 15	IPI00011302
P21589	NT5E	5'-nucleotidase ecto, CD73	12.2	14	0	2 of 15	IPI00009456
P04216	THY1	CD90, possible cell-cell or cell-ligand interaction	5.9	18	2	2 of 15	IPI00022892 (+1)
Q07021	C1QBP	Putative receptor for complement component 1 Q	5.8	6	0	6 of 15	IPI00014230
Q15758	SLC1A5	Sodium-dependent amino acids transporter	4.2	4	0	10 of 15	IPI00019472
P48960	CD97	CD97 antigen, isoform 2, potential cell adhesion receptor	3.4	3	0	1 of 15	IPI00299412 (+4)
P16070	CD44	CD44, Cell sruface receptor, cell-matrix interaction	2.8	22	7	8 of 15	IPI00297160 (+19)
P60033	CD81	CD81, Tetraspanin-28, cell surface protein	2.6	2	0	8 of 15	IPI00000190 (+1)
P29317	EPHA2, ECK	Ephrin type-A receptor 2, receptor tyrosine kinase	2.3	4	1	1 of 15	IPI00021267
GTPases; Other Signaling Proteins:							
P07947	YES	Src family tyrosine kinase Yes	6.6	7	0	6 of 15	IPI00013981
P11234	RALB	Ras-related protein Ral-B	5	5	0	5 of 15	IPI00004397
P10301	RRAS	Ras-related protein R-Ras	5	5	0	9 of 15	IPI00020418
P11233	RALA	Ras-related protein Ral-A	3.4	3	0	6 of 15	IPI00217519
P61226	Rap2b	Ras-related protein Rap-2b	3.4	3	0	6 of 10	IPI00018364
P04899	GNAI2	Ga(i)2, isoform 1, heterotrimeric G protein subunit	2.8	5	1	11 of 15	IPI00748145
P06241	FYN	Src familty tyrosine kinase Fyn, isoform 3	2.6	2	0	6 of 15	IPI00166845 (+2)
P08754	GNAI3	Ga(i)3, G(k) subunit alpha, heterotrimeric G protein subunit	2.6	2	0	11 of 15	IPI00220578
P07948	LYN	Src family tyrosine kinase Lyn, isoform B	2.6	2	0	3 of 15	IPI00432416 (+4)
Vesicular Trafficking:							
Q15836	VAMP3	VAMP3, Vesicle-associated membrane protein 3	30.6	37	0	11 of 15	IPI00549343
O75396	SEC22B	Vesicle-trafficking protein Sec22B	23.4	28	0	3 of 15	IPI00006865
P51809	VAMP7	VAMP7, Vesicle-associated membrane protein 7, isoform 1	9	10	0	8 of 15	IPI00020887
P51149	RAB7	Ras-related protein Rab-7a	6.6	7	0	4 of 15	IPI00016342
O14662	STX16	Syntaxin-16, Isoform B	6.6	7	0	1 of 15	IPI00023149 (+1)
P62820	RAB1A	Ras-related protein Rab-1A, isoform 1	5.8	6	0	5 of 15	IPI00005719 (+1)
P07355	ANXA2	Annexin A2, calcium/phospholipid-binding protein	5.8	6	0	6 of 15	IPI00418169 (+1)
O00161	SNAP23	SNAP23, Synaptosomal-associated protein 23	4.2	4	0	13 of 15	IPI00010438
P51148	RAB5C	Ras-related protein Rab- 5C	4.2	4	0	7 of 15	IPI00016339
O15126	SCAMP1	Secretory carrier-associated membrane protein 1	3.5	10	2	9 of 15	IPI00005129
P49755	TMP21	Transmembrane protein Tmp21	3.4	3	0	3 of 15	IPI00028055
O43760	SYNGR2	Synaptogyrin-2	3.4	3	0	2 of 15	IPI00013946 (+2)
O60499	STX10	Syntaxin 10	3.2	6	1	6 of 15	IPI00293402 (+1)

P61106	RAB14	Ras-related protein Rab-14	2.6	2	0	5 of 15	IPI00291928
O95292	VAPB	Vesicle-associated membrane protein-associated protein B/C	2.6	2	0	2 of 15	IPI00006211
P20340	RAB6A	Ras-related protein Rab- 6A	2.6	2	0	2 of 15	IPI00023526 (+1)
P61019	RAB2A	Ras-related protein Rab-2A	2.6	2	0	4 of 15	IPI00031169
Q99523	SORT1	Sortillin, as sorting receptor on Golgi compartment	2.5	7	2	5 of 15	IPI00217882
P04083	ANXA1	Annexin A1, calcium/phospholipid-binding protein	2.6	2	0	4 of 15	IPI00218918
O15400	STX7	Syntaxin-7, isoform 1	2	11	5	7 of 15	IPI00289876 (+1)
Cytoskeletal Proteins:							
P27105	BND7	Erythrocyte band 7 integral membrane protein	4.5	18	3	10 of 15	IPI00219682
P23528	CFL1	Cofilin-1, actin cytoskeleton regulation	3.4	3	0	5 of 15	IPI00012011
Q01628	IFM3	Interferon-induced transmembrane protein 3	3.4	3	0	7 of 15	IPI00303726
P07737	PFN1	Profilin-1, actin cytoskeleton regulation	2.6	2	0	5 of 15	IPI00216691
Scaffolding Proteins and Protein Folding:							
Q03135	CAV1	Caveolin, scaffolding protein within caveolar membranes	12.2	63	4	4 of 15	IPI00009236
P07237	PDIA1	Protein disulfide-isomerase PDI, protein folding	3.4	3	0	4 of 15	IPI00010796
P50990	CCT8	T-complex protein 1 subunit theta	3.4	3	0	5 of 15	IPI00302925 (+1)
P50991	CCT4	T-complex protein 1 subunit delta	2.6	2	0	4 of 15	IPI00302927 (+2)
P62937	PPIA	Peptidyl-prolyl cis-trans isomerase A, protein folding	2.6	2	0	7 of 15	IPI00419585
P30101	PDIA3	Protein disulfide-isomerase A3	2.6	2	0	3 of 15	IPI00025252
Q9Y287	ITM2B	Integral membrane protein 2B, amyloid precursor processing	2.6	2	0	7 of 15	IPI00031821
Metabolism:							
P04844	RPN2	RPN2, Subunit of N-oligosaccharyl transferase complex	10.6	12	0	4 of 15	IPI00028635 (+1)
P04843	RPN1	RPN1, Subunit of N-oligosaccharyl transferase complex	6.6	7	0	3 of 15	IPI00025874
P20674	COX5A	Cytochrome c oxidase subunit 5A, mitochondria	5	5	0	2 of 15	IPI00025086
P14618	PKM	Pyruvate kinase PKM, glycolysis	4.2	4	0	6 of 15	IPI00479186
P47985	UQCRFS1	Cytochrome b-c1 complex subunit 11, mitochondrial	4.2	4	0	3 of 15	IPI00026964
P53985	SLC16A1	Transporter for monocarboxylate, metabolism	2.8	8	2	2 of 15	IPI00024650
P31040	SDHA	Subunit of Succinate dehydrogenase complex, mitochondrial	2.6	2	0	3 of 15	IPI00965327
Q9NX63	CHCHD3	MICOS complex subunit MIC19, mitochondrial	2.6	2	0	5 of 15	IPI00015833 (+4)
Others							
Q7Z5G4	GOLGA7	GCP16, palmitoyltransferase, protein palmitoylation	5.8	6	0	9 of 15	IPI00480022 (+1)
Q6IAA8	LAMTOR1	Ragulator complex protein LAMTOR1, mTOR regulation	5	5	0	11 of 15	IPI00016670
Q96S97	MYADM	Myeloid-associated differentiation marker	5	5	0	5 of 15	IPI00102685 (+1)
Q04941	PLP2, A4	Proteolipid protein 2, function unknown	4.2	4	0	5 of 15	IPI00030362
Q96A26	FAM162A	Protein FAM162A	3.4	3	0	5 of 15	IPI00023001 (+1)
O95831	AIFM1	Apoptosis-inducing factor 1, Isoform 1, mitochondrial	2.6	2	0	2 of 15	IPI00000690 (+1)
P37235	HPCAL1	Hippocalcin-like protein 1, calcium binding and sensing	2.6	2	0	2 of 15	IPI00219344
Q56VL3	OCIAD2	Ovarian carcinoma immunoreactive antigen-like protein	2.6	2	0	1 of 15	IPI00555902

References

Liu, H., R.G. Sadygov, and J.R. Yates, 3rd. 2004. A model for random sampling and estimation of relative protein abundance in shotgun proteomics. *Analytical chemistry*. 76:4193-4201.

Old, W.M., K. Meyer-Arendt, L. Aveline-Wolf, K.G. Pierce, A. Mendoza, J.R. Sevinsky, K.A. Resing, and N.G. Ahn. 2005. Comparison of label-free methods for quantifying human proteins by shotgun proteomics. *Molecular & cellular proteomics : MCP*. 4:1487-1502.

Blanc, M., F. David, L. Abrami, D. Migliozzi, F. Armand, J. Burgi, and F.G. van der Goot. 2015. SwissPalm: Protein Palmitoylation database. *F1000Research*. 4:261.

Biology Open • Supplementary information
